# Observational evidence reveals the significance of nocturnal chemistry in seasonal secondary organic aerosol formation

**DOI:** 10.1038/s41612-024-00747-6

**Published:** 2024-09-06

**Authors:** Lu Liu, Thorsten Hohaus, Philipp Franke, Anne C. Lange, Ralf Tillmann, Hendrik Fuchs, Zhaofeng Tan, Franz Rohrer, Vlassis Karydis, Quanfu He, Vaishali Vardhan, Stefanie Andres, Birger Bohn, Frank Holland, Benjamin Winter, Sergej Wedel, Anna Novelli, Andreas Hofzumahaus, Andreas Wahner, Astrid Kiendler-Scharr

**Affiliations:** 1 Institute of Energy and Climate Research, IEK-8: Troposphere, Forschungszentrum Jülich GmbH, Jülich, Germany; 2 Laboratory of Atmospheric Chemistry, Paul Scherrer Institute, Villigen, Switzerland; 3 Department of Physics, University of Cologne, Cologne, Germany; 4 State Key Joint Laboratory of Environmental Simulation and Pollution Control, State Environmental Protection Key Laboratory of Atmospheric Ozone Pollution Control, College of Environmental Sciences and Engineering, Peking University, Beijing, China; 5 Environmental Research Centre, University College Cork, Cork, Ireland

**Keywords:** Atmospheric chemistry, Atmospheric chemistry

## Abstract

Oxidized Organic Aerosol (OOA), a major component of fine atmospheric particles, impacts climate and human health. Previous experiments and atmospheric models emphasize the importance of nocturnal OOA formation from NO_3_· oxidation of biogenic VOCs. This seasonal study extends the understanding by showing that nocturnal oxidation of biomass-burning emissions can account for up to half of total OOA production in fall and winter. It is the first to distinguish nocturnal OOA characteristics from daytime OOA across all seasons using bulk aerosol measurements. Summer observations of nocturnal OOA align well with regional chemistry transport model predictions, but discrepancies in other seasons reveal a common model deficiency in representing biomass-burning emissions and their nocturnal oxidation. This study underscores the significance of near-ground nocturnal OOA production, proposes a method to differentiate it using bulk aerosol measurements, and suggests model optimization strategies. These findings enhance the understanding and prediction of nighttime OOA formation.

## Introduction

Secondary organic aerosol (SOA) contributes substantially to atmospheric fine particles^1–3^, thus understanding SOA formation is essential to determining the effect of aerosols on climate^4^ and human health^5^. SOA is formed through the atmospheric oxidation of volatile organic compounds (VOCs) emitted from both biogenic and anthropogenic sources^6^. However, the exact formation and evolution process of SOA in the atmosphere is still uncertain and hence limits the predictability of aerosol concentrations and therefore their effects^7^.

In field studies, the concentration of oxidized organic aerosol (OOA)^1,7^ resolved by receptor models^8,9^ from the measurement of total organic aerosols (OA) by instruments like the aerosol mass spectrometer (AMS), are commonly used to show the OA contribution from secondary sources differing from primary emissions. Receptor models can further divide OOA into subtypes. Subsequently, the chemical evolution of ambient OA can be analyzed using changes in the properties of the resolved OOA subtypes with regard to volatility^1,10,11^ and oxidation degree^12–14^.

Nocturnal oxidation of biogenic VOCs has been shown to form significant OOA in chamber experiments^15,16^. Global chemistry transport models predict that OOA formation from nighttime oxidation of biogenic VOCs by the nitrate radical (NO_3_·) accounts for 5% to 21% of the global SOA production^17,18^. However, in the interpretation of field study results, the formation and aging of OOA are still considered to be driven by mainly photochemistry^1,19^, even though unexplained concentration increases of OOA during the night have been observed in many ground-based field studies^9,20–23^.

In addition, in the study by Kiendler-Scharr et al. ^24^, NO_3_-initiated oxidation of biogenic VOCs during nighttime has been shown to be the major source of significant particulate organic nitrate in Europe and has been estimated to be a ubiquitous and important contributor to submicron aerosol mass on the continental scale. However, in that study, the source of organic nitrate observed during cold seasons remained unresolved, suggesting a significant gap in the understanding of nocturnal particle formation. This knowledge gap may also be a contributing factor to why current chemical transport models systematically underpredict the observed OOA concentrations during winter, especially in Europe^25^. A recent chamber study by Kodros et al. ^26^ found that OOA was rapidly formed through NO_3_· oxidation of organic compounds emitted from biomass-burning. Moreover, in their study, the mass of OOA formed from the oxidation process was comparable to the amount of organic aerosol emitted directly from biomass-burning. However, such a large OOA source especially in wintertime still lacks direct evidence from field studies.

Biomass-burning emissions are globally increasing due to more frequent wildfire activity in a warming climate^27,28^. In addition, it has been reported that biomass-burning, such as residential heating, is increasing e.g. in Europe^29^ contributing significantly to particulate pollution in densely populated areas during winter^30^. Also, due to the energy crisis triggered by recent military conflicts, there is a shift from using fossil fuels for heating systems towards using more electric or biomass-fuel alternatives in Europe^31^. Therefore, fresh and chemically aged aerosol from biomass-burning sources may gain further importance in the future.

During the Jülich Atmospheric Chemistry Project (JULIAC) campaign^32,33^ (Supplementary Note 1), the concentrations of atmospheric components, including oxidants, trace gases, and the chemical composition of submicron aerosol (Supplementary Fig. 1), were measured in the atmospheric simulation chamber SAPHIR^34,35^ in Jülich. The chamber was continuously flowed with ambient air sampled from a height of 50 m extending well above the canopy and buildings in the vicinity of the chamber. The air had a residence time of 1 h in the chamber. By utilizing the comprehensive dataset and combining it with the analysis of the chemical composition of aerosols by a receptor model, Positive Matrix Factorization (PMF; Methods), we investigated the seasonality of the nocturnal formation of OOA. This study showed that the nocturnal oxidation of organic species is as important as their photo-oxidation for OOA production throughout all seasons. The characteristics of the chemical composition and the diurnal changes in concentration of nocturnal OOA determined from our measurements can be used for identifying nocturnal OOA in other field studies. Furthermore, a comparison between the observed OOA concentrations attributed to nighttime oxidation and results from the European Air pollution Dispersion–Inverse Model (EURAD-IM, Methods), reveal that the pathway of nocturnal oxidation is not well represented in the EURAD-IM model during all seasons except summer.

## Results and Discussion

### Ambient observation of large OOA formation from nocturnal chemistry

**Fig. 1:**
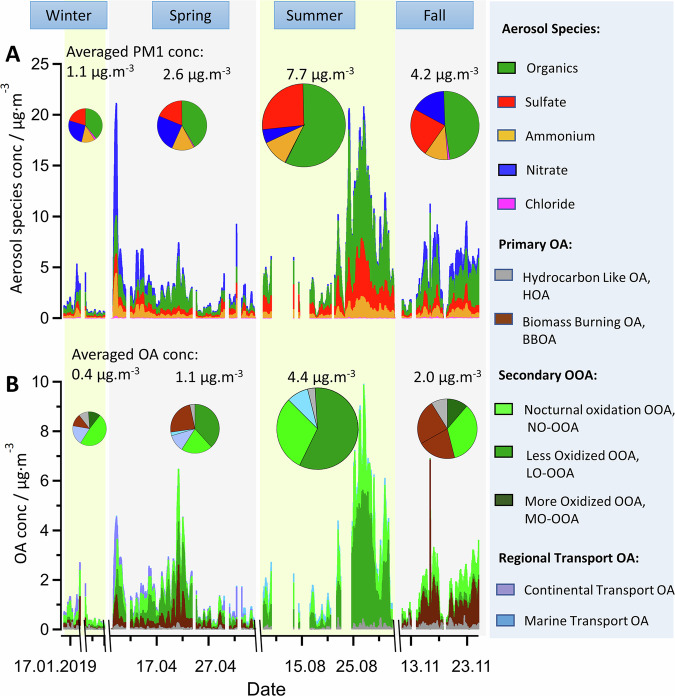
Seasonal overview of sources of organic aerosol and their contribution to submicron aerosol mass. Time series of aerosol concentrations and average values during the four seasons of the JULIAC campaign for (**A**) the chemical composition of submicron aerosol, and (**B**) directly emitted organic aerosol (primary OA), secondary organic aerosol formed from oxidation processes (OOA) and organic aerosol from regional transport obtained from a PMF analysis of the measured mass spectrum of organic aerosol.

**Fig. 2:**
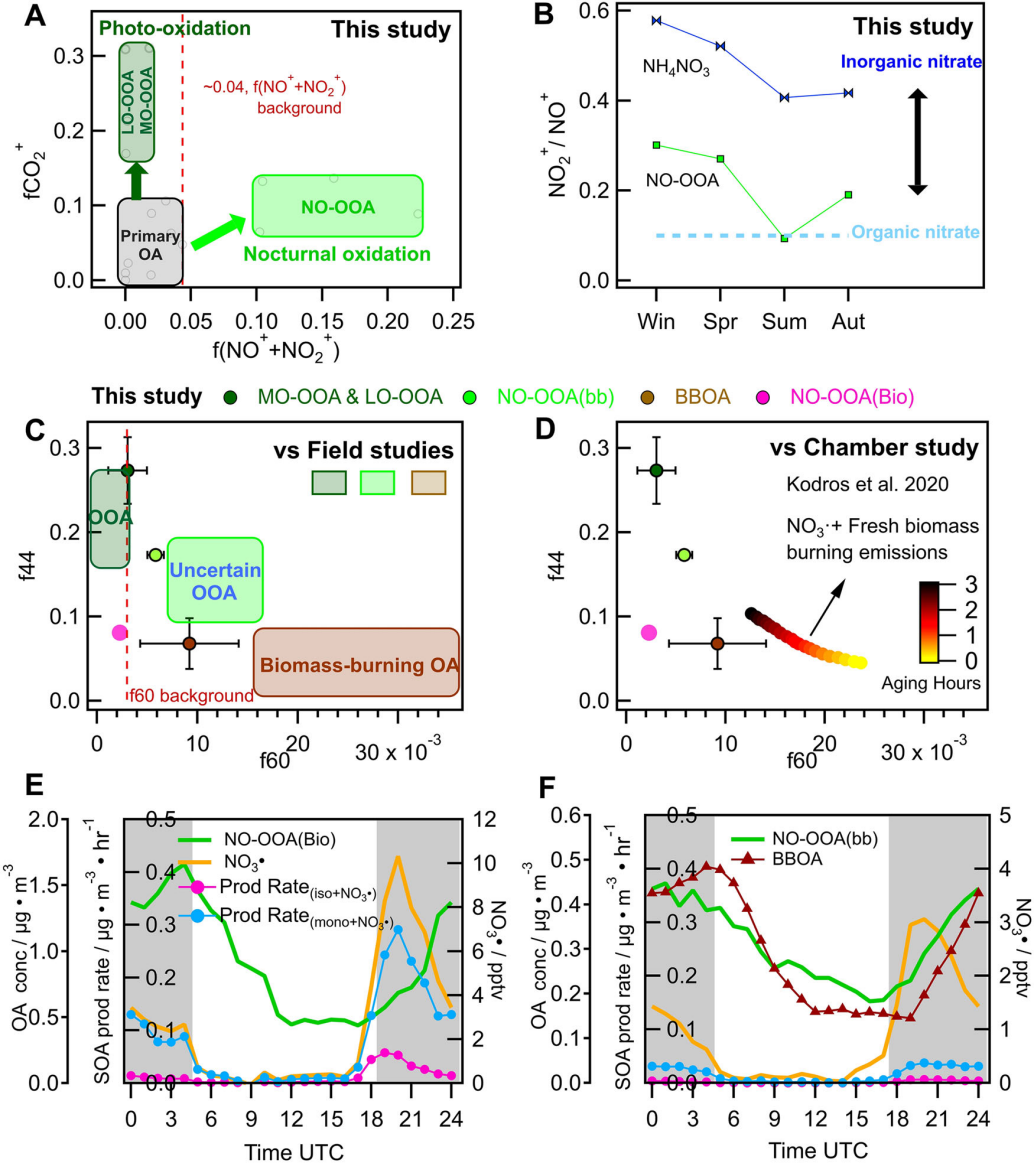
Analysis of chemical characteristics and formation mechanism of nocturnal OOA. **A** Directly emitted primary OA, OOA formed from photo-oxidation (LO-OOA and MO-OOA) and nocturnal oxidation processes (NO-OOA) resolved by the PMF analysis of particulate nitrate and organic aerosol measurements during the JULIAC campaign. The ratio of the ion mass signals of the fragments CO_2_^+^ (fCO_2_^+^) vs the sum of NO^+^ and NO_2_^+^ (f(NO^+^ + NO_2_^+^)) normalized to the total ion mass signal intensity is used to show the distribution of nitrate-containing OA factors. These OA factors fall in fCO_2_^+^ vs f(NO^+^ + NO_2_^+^) space and are represented by gray circles and categorized by the grey, dark green, and light green rectangles respectively, with a detailed graph in Supplementary Fig. 25. The value of f(NO^+^ + NO_2_^+^) (<0.04) of primary OA and photo-oxidation OOA is marked as background by a red dashed line. **B** The ratio of nitrate fragments (NO_2_^+^/NO^+^) in NO-OOA across seasons is shown by green markers with line, while blue markers with line represent the ratio of inorganic ammonium nitrate particles (NH_4_NO_3_). The blue dashed line represents the reference ratio of pure organic nitrate^24^. **C** The chemical composition, characterized mass spectrum characteristics of f44 vs f60, is used to compare the averaged PMF factors from this study (shown as circle with error bar) to those from previous field studies^9,20,52,53^ (depicted as rectangles). These studies all identified an unexplained nocturnal OOA, daytime OOA, and primary BBOA, highlighted by light green, dark green, and brown rectangles, respectively. Detailed factor positions are provided in Supplementary Fig. 25. The background value of f60 (~0.3%) for atmospheric OA^87,88^ is marked by a red dashed line. Additionally, (**D**) compares the averaged OA factors obtained in this study to the OA evolution during the dark oxidation of biomass-burning emission by NO_3_· in a chamber study^87,88^, illustrated with dotted line color-coded by aging time. The seasonal averaged diurnal pattern of the mass concentration of the NO-OOA, BBOA, NO_3_·, and SOA production rate of the NO_3_· reaction with monoterpenes and isoprene are shown for summer (**E**) and spring (**F**). The grey areas represent nighttime.

The chemical composition of non-refractory submicron particles, mainly aerosol constituents (organics, nitrate, sulfate, chloride, and ammonium) was measured by a high-resolution time-of-flight aerosol mass spectrometer (HR-ToF-AMS) during four intensive JULIAC episodes in each season in 2019 (Fig. 1). Overall, organic compounds were found to be the major components of measured submicron aerosol throughout the year accounting for 40% to 60% of the total aerosol mass in this study. The PMF analysis attributed the measured organic aerosol to (1) direct emissions (primary organic aerosol) of traffic exhaust (HOA) and biomass-burning (BBOA), (2) formation from oxidation products (OOA), and (3) long-range regional transport (Supplementary Figs. 2–5). In the PMF analysis, OOA is typically resolved into two subtypes that differ in their volatility and degree of oxidation^12,14,36^ and both are considered to originate mainly from photochemical activities with maximal concentrations during daytime. However, in this study, in addition to the two common subtypes of OOA from photo-oxidation, less-oxidized OOA (LO-OOA), and more-oxidized OOA (MO-OOA), a third subtype of OOA was resolved. The third OOA subtype is shown to mainly originate from nocturnal oxidation and is therefore labeled as nocturnal oxidation OOA (NO-OOA) in this work. The PMF analysis of only aerosol organics concentrations is commonly applied but fails to differentiate between OOA formed from nighttime oxidation and photo-oxidation. The reason is that, in bulk aerosol measurements, the mass spectrum of OOA becomes increasingly similar with higher levels of oxidation, regardless of whether the oxidation occurs during daytime or nighttime. Therefore, the PMF analysis was applied including both the nitrate and organics of aerosol (Methods). This allows us to distinguish between the types of OOA, as a larger nitrate fraction (NO^+^ + NO_2_^+^ fragments in the mass spectrum) (Fig. 2A) and a higher nitrogen-to-carbon ratio (N:C, Supplementary Fig. 7) is obtained for NO-OOA than for OOA from photo-oxidation.

**Fig. 3:**
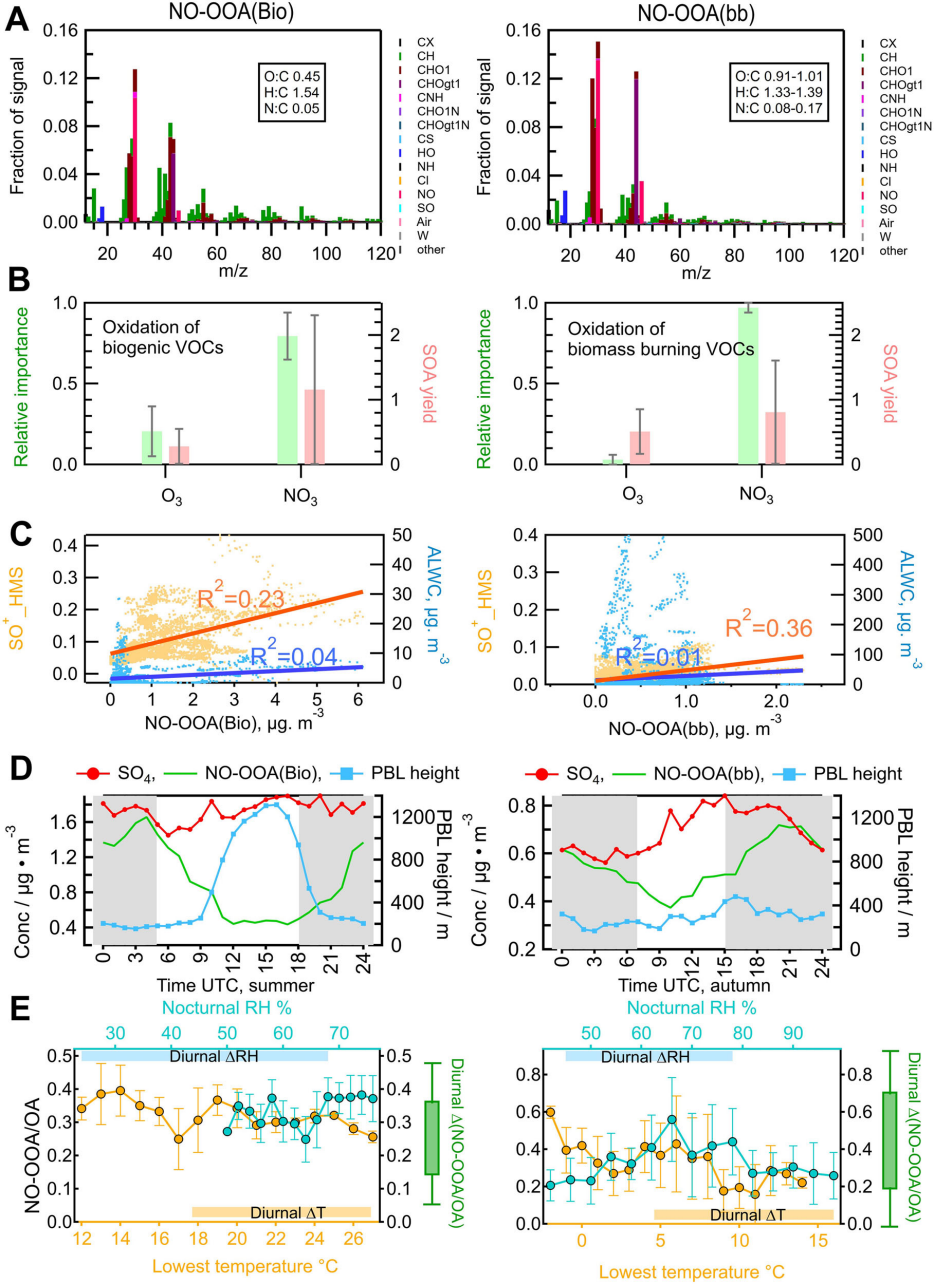
Analysis of atmospheric layer development, phase-partitioning, and chemical reactions on the enhancement of nocturnal OOA. **A** The ion mass spectrum of NO-OOA(Bio) for summer and an averaged spectrum of NO-OOA(bb) for the other seasons (detailed spectra in the Supplementary Fig. 7) obtained by the PMF analysis of measurements of aerosol nitrate and organics. **B** Competition between NO_3_ and O_3_ oxidation of VOCs (label as relative importance (green)) and the comparison of secondary organic aerosol (SOA) yield of biogenic VOCs (BVOCs) (isoprene, α-pinene, β-pinene, and limonene) and biomass-burning volatile organic compounds (bbVOCs) (furan, naphthalene) by different oxidants (NO_3_ and O_3_) (red), with the median values represented by the bars and the range of variability indicated by the error bars. (Supplementary Table 2). **C** Correlation of NO-OOA(Bio) and NO-OOA (bb) with the SO^+^ fragment originating from HMS and aerosol liquid water content (ALWC). **D** Averaged diurnal variations of the sulfate (SO_4_) aerosol mass concentrations and observed NO-OOA obtained by the PMF analysis, as well as the PBL height obtained from the EURAD-IM in summer and autumn during the JULIAC campaign. Plots showing results for the other seasons can be found in the Supplementary Fig. 18. **E** The variation of mass ratio of NO-OOA (Bio) to total OA in summer, and NO-OOA (bb) to total OA in the other seasons as a function of the minimum nocturnal temperature and corresponding relative humidity during these periods. Data is restricted to the period ±2 hours of the minimum temperature. The averaged diurnal variations of temperature, RH, and the ratio of NO-OOA/OA are also displayed in the plot by yellow, blue, and green bars, respectively.

In our study, the exact mass spectrum of NO-OOA measured by the AMS instrument varied between the different seasons (Supplementary Fig. 7) due to different dominant precursors being mainly biogenic VOCs in summer and being compounds emitted from biomass-burning in the other seasons. Therefore, NO-OOA was designated as NO-OOA(Bio) for summer and NO-OOA(bb) for the other seasons (Fig. 3A). The NO-OOA(Bio) has a lower degree of oxidation, indicated by an elemental oxygen to carbon ratio (O:C) of 0.39. Additionally, it shows a low intensity of the characteristic ion mass signal for levoglucosan at mass-to-charge ratio (m/z) 60, which is only 0.2%. In contrast, NO-OOA(bb) shows a higher oxidation degree, with an O:C ratio ranging from 0.76 to 0.91 and a higher intensity ratio of the signal at m/z 60 of 0.5–0.7%. Additionally, the concentrations of both NO-OOA(Bio) and NO-OOA(bb) in the different seasons show consistently a peak value during nighttime hours. This nocturnal peak well explains the observed increase in the overall oxidation degree of the submicron organic aerosols (OA) at night (Supplementary Fig. 8). In our study, NO-OOA constituted 20% to 50% of the total submicron OA mass with the highest average concentrations of 1.3 μg m^−3^ in summer. During this period, concentrations of NO_3_· were also at their peak (derived from measured dinitrogen pentoxide (N_2_O_5_) and nitrogen dioxide (NO_2_), see Supplementary Fig. 1). This concurrent seasonal peak supports that NO-OOA formation is driven by NO_3_· chemistry. During summer, NO-OOA contributed around 30% of the overall mass of OOA produced. This is slightly higher than the value of 5% to 21% predicted in previous global model studies^17,18^. In contrast, during winter and fall, NO-OOA became a major part of OOA, demonstrating the importance of nocturnal chemistry for ambient OOA formation and aging during cold seasons at this semi-rural site.

### Nocturnal OOA formation via the NO_3_-initiated oxidation of precursors in different seasons

Previous studies have reported that NO_3_-initiated oxidation in the atmosphere is commonly accompanied by a significant enhancement of particulate organic nitrate concentrations^24,37^. In this study, NO-OOA resolved from the PMF analysis of measured aerosol organics and nitrates (Supplementary Figs. 9–12), contained high concentrations of organic nitrates (Methods). This is shown by the molar ratio of the ion fragments NO_2_^+^ to NO^+^ in NO-OOA being lower than in ammonium nitrate (Fig. 2B), demonstrating that NO_3_-initiated nocturnal oxidation significantly contributed to the enhancement of NO-OOA.

The analysis of the competition between NO_3_· and O_3_ (Methods) shows that NO_3_· was the dominant oxidant in the night in this study (Fig. 3B). To estimate the potential effect of aqueous phase chemistry on the formation of NO-OOA, the ion mass signal of the sulfate fragment (SO^+^) presumably from hydroxymethanesulfonate (HMS), a tracer for liquid phase chemistry^38^, was calculated by ion fragmentation method^39^. The time series of NO-OOA showed a lack of correlation with both the sulfate fragments from particulate HMS and the aerosol liquid water content (ALWC)^40,41^ for most of the observations (Fig. 3C). In addition, aqueous chemistry was unlikely important in this study, as the aerosol contained little water (ALWC < 10 μg/m^3^) for most of the time in this campaign (Supplementary Table 3). Only during the cold season, NO-OOA concentrations weakly correlated with sulfate fragments from particulate HMS (*R*^2^ = 0.36), and a concurrent increase in the ALWC and the concentration ratio of NO-OOA(bb)/BBOA was also observed (Supplementary Fig. 13). Therefore, a small contribution of aqueous and heterogeneous reactions of NO_3_· and N_2_O_5_^42^ to NO-OOA cannot be completely excluded during the cold seasons (winter, spring, and fall).

In winter, spring, and fall, the high concentrations of NO-OOA(bb) observed were produced mainly from the NO_3_· oxidation of biomass-burning emissions. This is evident, as NO-OOA(bb) correlated with primary organic aerosol emitted by biomass-burning emissions (BBOA, *R*^2^ 0.48–0.62), as well as with gas-phase tracers for biomass-burning such as furan (*R*^2^ 0.32–0.49) and CO (*R*^2^ 0.44–0.75), and the characteristic ion mass signal (mass to charge ratio, m/z 60, C_2_H_4_O_2_^+^, *R*^2^ 0.68–0.82) from levoglucosan, which is regarded as a tracer for biomass-burning in particles (Supplementary Table 6). Moreover, the changes in the OA composition measured in a chamber study^26^ of fresh biomass-burning emissions oxidized by NO_3_· (Fig. 2D) showed a decrease of the ion mass signal at m/z 60 accompanied by an increase of the ion mass signal at m/z 44 (an indicator for aerosol aging, mainly CO_2_^+^) with increasing aging. The same behavior is also observed in this study for BBOA and NO-OOA(bb) (Fig. 2D), which further supports that biomass burning is the precursor of NO-OOA(bb). In addition, the OOA produced in the chamber study showed a similar mass spectrum as observed in NO-OOA(bb) in the JULIAC campaign, characterized by a high linear correlation coefficient of *R*^2^ = 0.94 and a theta angle^43^ of *θ* = 14.0° (Supplementary Fig. 14). In addition, the diurnal variations and concentrations of BBOA and NO-OOA(bb) are very similar in this study (Fig. 2F), suggesting a common source. This is consistent with the model prediction in Kodros et al.^26^, where ~60–70% of OA related to biomass-burning emissions were found to be affected by NO_3_· nighttime chemistry. It is important to note that boundary layer dynamics can also affect the diurnal distribution of OOA, as discussed in the next section.

In summer, the formation of NO-OOA(Bio) was dominated by the NO_3_-initiated nocturnal oxidation of biogenic VOCs, especially monoterpenes. This is supported by the similarity of the chemical composition (*R*^2^ = 0.64–0.71, *θ* = 27.8°–34.3°) of NO-OOA(Bio) observed in this study and the OOA produced from the NO_3_-initiated oxidation of monoterpenes (β-pinene and limonene) in a previous chamber experiment^44^ (Supplementary Fig. 14). In addition, the aerosol production rates calculated from the rate constant of NO_3_· reactions with isoprene and monoterpenes and the aerosol yields (Methods) support this conclusion, as the NO_3_· oxidation of monoterpenes gave a higher SOA production rate than isoprene (Fig. 2E). The calculated total SOA concentration produced via NO_3_· oxidation of monoterpenes during the night (~1.7 µg m^−3^) was even comparable to the average nocturnal enhancement of NO-OOA(Bio) (~1.2 µg m^−3^). Overall, these findings demonstrate that the NO_3_-initiated oxidation of monoterpenes was the main contributor to the enhancement of nocturnal OOA during the JULIAC campaign in the summer.

### Non-dominant role of atmospheric layer development and phase partitioning in nocturnal OOA

In addition to the chemical production, organic aerosol concentrations near the ground can be affected by vertical mixing during the development of atmospheric layers. Specifically at night, the vertical mixing is often poor. In this campaign, the sampling point was at a height of 50-m, which was above the surface layer ( 30 m height) for most of the time and, therefore, located in the nocturnal boundary layer (Methods and Supplementary Fig. 15). The vertical distribution of SOA formed from NO_3_· oxidation (NO_3_-SOA) during the JULIAC campaign (Supplementary Figure 16) was simulated by the regional chemistry transport model EURAD-IM (Methods) to estimate the vertical mixing of particles. The simulation shows a clear nocturnal increase of NO_3_-SOA concentrations at the ground, indicating a significant SOA production from nighttime chemistry rather than an accumulation of particles. In addition, the weak correlation (*R*^2^, 0.12–0.22) between the planetary boundary layer (PBL) height and the modeled NO_3_-SOA concentrations from NO_3_· oxidation at a height around 50 m, further confirms that particulate accumulation in the nocturnal boundary layer was not a driving factor for the observed increase of nocturnal OOA. This conclusion is further supported by a much smaller enhancement of low-volatile particulate sulfate measured by the HR-ToF-AMS instrument during the night when NO-OOA increased as observed in all seasons (Fig. 3D).

Furthermore, phase partitioning of pre-existing semi-volatile components in the gas phase driven by the changes in diurnal temperature and relative humidity (RH), also could promote the nocturnal formation of OA^45^. Aerosol bulk nitrate measured by the HR-ToF-AMS instrument is commonly used as a tracer for the volatile aerosol components, based on its volatility^46^ and atmospheric lifetime (~7.6 days)^47^. A weak correlation between NO-OOA and aerosol bulk nitrate (*R*^2^, 0.28, Supplementary Table 6) was observed during the JULIAC campaign. In addition, the variation in the mass fraction of NO-OOA to total OA (NO-OOA/OA), as a function of the lowest night-time temperatures and corresponding RH (Fig. 3E), demonstrates the effect of phase-partitioning in NO-OOA concentration. No consistent increase in NO-OOA/OA with a rise in nocturnal RH was observed, suggesting a weak or negligible RH-dependent gas-particle partitioning^48^. Meanwhile, a slight increase in NO-OOA/OA with a decrease in temperature was found, but that increase can be attributed to the phase-partitioning of both pre-existing volatile compounds and fresh volatile products from nocturnal chemistry. Despite this, the increase in NO-OOA/OA corresponding to average diurnal temperature variation does not reach half of the overall average diurnal change in NO-OOA/OA. In addition, when temperatures drop below 0 °C, an increase in biomass burning emissions was observed (Supplementary Fig. 17), likely due to increased residential heating. This could also explain the stronger temperature-dependent increase in NO-OOA(bb)/OA during the colder seasons. Therefore, we concluded that the phase partitioning of pre-existing volatile compounds may contribute to the nocturnal enhancement of NO-OOA, but it is not a dominant factor.

During the day, the concentrations of NO-OOA decreased due to the combined effects of dilution by vertical mixing during the development of the PBL, evaporation of volatile compounds due to the temperature increase, and aerosol aging by photo-oxidation processes. On most days, aerosol sulfate increased in the morning (Fig. 3D), indicating the mixing of air masses with sulfate-rich aerosol from the residual layer into the newly formed PBL. This vertical mixing during the daytime PBL formation can also dilute the NO-OOA concentration observed near the ground. The concurrent decrease of the aerosol nitrate and NO-OOA concentrations during daytime was accompanied by an increase in the more-oxidized OOA concentrations (Supplementary Fig. 19), implying a combined effect of evaporation of volatile compounds and aerosol aging.

### Significant organic aerosol formation via nocturnal oxidation is ubiquitous

Unexplained significant enhancements of OOA during nighttime have been frequently observed in previous field studies^9,13,21,22,38,49^, indicating the ubiquity of nocturnal oxidation in the atmosphere. Potential OOA formation from nocturnal oxidation might have been underestimated and potentially subsumed into other OA types such as BBOA, OOA from photo-oxidation, or OOA subtypes with uncertain origin in the PMF analyses (Supplementary Table 4). The comparison of aerosol mass spectra observed in previous studies^13,20,21,50,51^ and NO-OOA(bb) or NO-OOA(Bio) shows that OOA types from unspecific sources had similar compositions as NO-OOA(bb) (*R*^2^ = 0.81-0.94, *θ* = 13.7°-24.1°) or NO-OOA(Bio) (*R*^2^ = 0.78–0.96, *θ* = 14.3°–27.7°) derived in this work (Supplementary Figs. 20 and 21). In some of the previous studies^9,20,52,53^, which resolved both primary BBOA and unspecific nocturnal OOA, the aerosol composition with regard to the ratio of the integrated ion mass signal at m/z 44 and m/z 60 to the total ion mass signal (f44 and f60) shows a similar chemical composition and mass spectrum characteristics compared to the nocturnal oxidation of biomass-burning emission observed in this study (Fig. 2C and Supplementary Fig. 25). Hence, our results indicate that significant nocturnal OOA formation is ubiquitous. Additionally, the main characteristics of OOA formed from nocturnal oxidation at different seasons determined in this study could help identifying and quantifying the nocturnal OOA production in future observations.

### Model prediction of SOA from NO_3_· oxidation across Europe

**Fig. 4:**
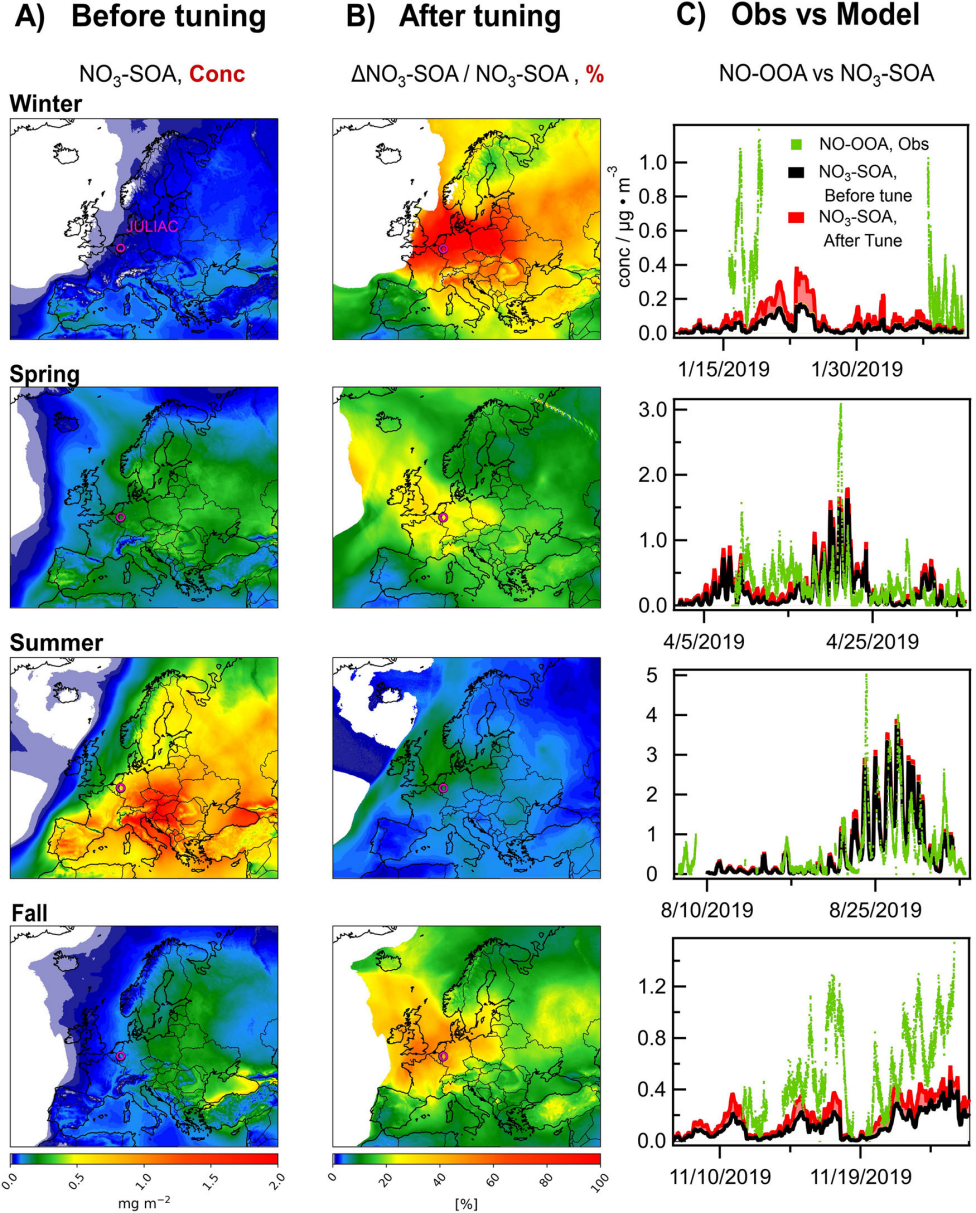
Model prediction and optimization of SOA from NO_3_· oxidation across Europe. **A** The seasonally averaged and vertically integrated column density of secondary organic aerosol from NO_3_· oxidation (NO_3_-SOA) across Europe predicted by the EURAD-IM model, and (**B**) their relative increase of vertically integrated column density for optimized emissions and aerosol yields (ΔNO_3_-SOA/ NO_3_-SOA). Panel **C** showed a comparison of the concentrations of secondary organic aerosol from nighttime oxidation in the observations (NO-OOA) and the EURAD-IM (NO_3_-SOA) before and after optimization at around 50-m height at the JULIAC site (marked by the red circle in **A** and **B**).

The SOA formed from NO_3_· oxidation (NO_3_-SOA) over Europe was calculated by the EURAD-IM (Methods). In the model, the concentration of NO_3_-SOA is calculated based on known reaction kinetics and SOA yields of organic compounds. The EURAD-IM predicted the highest NO_3_-SOA concentrations in central and southern Europe, particularly at low altitudes (Supplementary Fig. 16), showing the ubiquity of nocturnal oxidation at the near-ground levels across Europe (Fig. 4A), mainly during summer. At the JULIAC measurement site, the time series of observed NO-OOA concentrations and NO_3_-SOA concentrations calculated by the EURAD-IM show a similar behavior in all seasons (*R*^2^ ranging from 0.50 to 0.86). During summer, when biogenic VOCs emissions were high and NO_3_· oxidation of biogenic VOCs was the dominant source for nocturnal OOA formation as determined from the observations, the agreement between model predictions and measurements was strongest and characterized by similar shapes of the time series and comparable concentrations (Fig. 4C). Therefore, our measurements give evidence that the nocturnal formation of SOA from the oxidation of biogenic emissions is well represented in the EURAD-IM.

In contrast, in spring, fall, and winter when measurements showed that the oxidation of biomass-burning emissions was the dominant path for the nighttime formation of OOA, concentrations of NO_3_-SOA calculated by the EURAD-IM were 2 to 10 times lower than the measurements. This discrepancy indicates that this formation pathway is not well represented in the EURAD-IM. In the model, most of the NO_3_-SOA is produced from the NO_3_· oxidation of isoprene and monoterpenes (Supplementary Fig. 22). VOCs from biomass-burning emissions such as phenolic compounds are not included in the emission inventory used in most models^54^, although the NO_3_· oxidation of these compounds is potentially significantly contributing to the nighttime formation of SOA^42,55^. Moreover, compared to the results of recent laboratory studies^56,57^, a quite low SOA yield ( ~ 0.02) of oxidation products of VOCs from biomass burning is implemented in the aerosol dynamics module of the EURAD-IM. Model sensitivity runs (Methods, Supplementary Fig. 23) show that including emissions of three phenolic compounds typical for residential heating and implementing a lower limit SOA yield of 0.3, increases the modeled NO_3_-SOA concentrations by about a factor of two in all areas in Europe during the cold seasons (Fig. 4B). This shows that the underestimation of biomass-burning in the model could explain the discrepancy between observed and modeled SOA formed from nighttime chemistry during the cold season. This underestimation is likely present in most chemistry transport models. Therefore, revised emission inventories and SOA yields are recommended to ensure an accurate representation of these processes in models.

In this work, the seasonal characteristics and the chemical composition of aerosols from the NO_3_-initiated oxidation of organic compounds were determined for the first time. Results can be widely applied in the analysis of field studies to distinguish nocturnal organic aerosol using a PMF analysis. The ubiquity of the aerosol formation from nocturnal oxidation across Europe is shown by calculations using the chemistry transport model EURAD-IM. Measurements in this study also show that the NO_3_· oxidation of organic compounds from biomass burning was the dominant source for the formation of secondary aerosol in the cold seasons, but this source is not well represented in current chemical transport models. Considering the future increase in biomass-burning emissions, the nocturnal oxidation of these compounds is expected to gain in importance. Therefore, further studies are required to accurately represent secondary aerosol formation from nocturnal chemistry in models.

## Methods

### The JULIAC campaign

The JULIAC campaign (Supplementary Note 1) took place at the semi-rural site on the campus of the Forschungszentrum Jülich (50.91 N, 6.41E), North Rhine-Westphalia, Germany from January to November 2019. During the campaign, ambient air was continuously sampled through an inlet mounted on a 50m-high tower and injected into the atmosphere simulation chamber SAPHIR^34,35,58^. At night, the sampling height was mostly above the nocturnal surface layer (averaged height of 30 m). Air masses observed during the JULIAC campaign could have been affected by anthropogenic emissions from the nearby city Jülich with industry (distance <5 km) and by biogenic emissions from a nearby forest (distance <1 km) (Supplementary Fig. 24). The inlet was above the canopy height of the surrounding forest. A comprehensive set of instruments (Supplementary Note 2) was used to analyze the air in the SAPHIR chamber. The well-mixed air in the chamber ensured that all instruments observed the same air composition.

### Positive Matrix Factorization (PMF) of non-refractory aerosols measured by the HR-ToF-AMS

The chemical composition of non-refractory submicron aerosols was measured by HR-ToF-AMS. The ion mass signals were analyzed using a receptor model, PMF, in order to attribute sources of aerosols (Supplementary Note 3). PMF is a mathematical technique to treat bilinear unmixing problems^59^ and has been extensively applied in aerosol source apportionment studies^1,8,12,60,61^. In this study, the software Source Finder (SoFi Pro 8.0.3.1) was used to analyze the contributions of the different sources to the aerosol^62,63^. The optimal solutions of the PMF analysis were defined based on the residuals, factor features (e.g., tracer ions, diurnal pattern), and the interpretability of the factor’s time series with tracer quantities (VOCs, radicals, photolysis frequencies, wind directions, wind speeds, etc.). The determined source factors for the organic fraction of the aerosol from one season were constrained and taken as prior factors for the PMF analyses when the nitrate fraction was included. A potential artificial bias introduced by including the nitrate fraction in the PMF analysis can be excluded, as the PMF results with and without including the nitrate fraction were similar (Supplementary Table 5). The robustness of the PMF results was explored by a statistical analysis of 200 bootstrap runs performed by constrained PMF analysis with the random a-values method (Supplementary Figure 6). The elemental ratios for all factors were calculated based on the improved ambient method by Canagaratna et al. ^64^.

### Calculations of NO_3_· concentrations, loss of VOC by oxidation with NO_3_· and O_3_ and SOA production rate of NO_3_· oxidation

Nitrate radical (NO_3_·) concentrations were calculated from measured N_2_O_5_ and NO_2_ concentrations using their thermal equilibrium^65^. The competition between the oxidation of VOC by NO_3_· and O_3_ (denoted relative importance) is used to describe the significance of NO_3_· for the oxidation of one species of VOCs (marked as species i)^13^. The value of averaged nighttime (UTC 18:00- 5:00, Day+1) NO_3_·, O_3,_ and VOC concentrations, and calculated temperature-dependent reaction rate constants (Supplementary Table 1, NIST kinetics database https://kinetics.nist.gov/kinetics/KineticsSearchForm.jsp) were used for calculation.

1 $${{Relative\; importance}}_{{species\; i}+N{O}_{3}{{\cdot }}}=\frac{{k}_{\left[{species\; i}+N{O}_{3}{{\cdot }}\right]}\times [N{O}_{3}{{\cdot }}]}{{k}_{\left[{species\; i}+N{O}_{3}{{\cdot }}\right]}\times [N{O}_{3}{{\cdot }}]+{k}_{\left[{species\; i}+{O}_{3}{{\cdot }}\right]}\times [{O}_{3}]}$$

A 10% SOA yield for the NO_3_·oxidation products of isoprene was used based on previous studies giving SOA yields between 2% and 15%^15,37,66^. The SOA yield for NO_3_· oxidation products of monoterpenes significantly varies for different monoterpene species (Supplementary Table 2). Assuming that α-pinene (SOA yield, 0.7–25%)^67–69^ was the most abundant monoterpene, followed by β-pinene (SOA yield, 5–55%)^13,16,44^ and limonene (44–231%)^44,70^, a lower limit for the SOA yield of 20% for NO_3_· oxidation products of monoterpenes was used in this work.

### Calculations of the fraction of particulate organic nitrate

The fraction of particulate organic nitrate was determined from the relative ion mass signals of the NO_2_^+^ and NO^+^ fragments (*R*_measured_) detected by the HR-ToF-AMS instrument following the fragment pattern approach^24,71^:

2 $${pOrgN}{O}_{3,{frac}}=\frac{(1+{R}_{{OrgNO}3})\times ({R}_{{measured}}-{R}_{{calib}})}{(1+{R}_{{measured}})\times ({R}_{{OrgNO}3}-{R}_{{calib}})}$$

The ratio of NO_2_^+^ to NO^+^ ion mass signals for pure inorganic nitrate (*R*_*calib*_) was determined from calibration measurements of the HR-ToF-AMS instrument with ammonium nitrate particles. In this study, a ratio of 0.1 for pure organic nitrate (*R*_*OrgNO3*_)^24^ was used to calculate the concentrations of particulate organic nitrate.

### Estimation of atmospheric layer heights

In this study, the heights of near-ground atmospheric layers were determined from the vertical profile of the potential temperature ($\theta$). The potential temperature was calculated from the ambient temperature measurements at different heights between 2 m and 120 m on a tower close to the measurement site (Supplementary Fig. 15). A positive change in the potential temperature with height implies a stable atmosphere, whereas a negative change indicates an unstable or well-mixed atmosphere^72,73^.

### The EURAD-IM

The regional chemistry transport model EURAD-IM (European Air pollution Dispersion–Inverse Model)^74–76^ was used to simulate atmospheric trace gas and aerosol concentrations in Europe during the JULIAC campaign. The dynamics within the EURAD-IM simulations are driven by meteorological forecasts using the Weather Research and Forecasting Model (WRF Version 3.7)^77^. Boundary conditions were extracted from the CAMS global reanalysis EAC4^78^ for atmospheric constituents and the ERA5 reanalysis for meteorology^79^. The EURAD-IM includes anthropogenic as well as biogenic emissions of trace gases and aerosols. Anthropogenic emissions^80^ represent 2011 data for NH_3_, CO, NO_x_, SO_x_, NMVOCs, PM10, and PM2.5. Emissions of biogenic VOCs were calculated by the Model of Emissions of Gases and Aerosols from nature (MEGAN) V2.1^81^. In EURAD-IM, the aerosol dynamics are simulated by the Modal Aerosol Dynamics Model for Europe (MADE)^82^ with the Secondary ORGanic Aerosol Model (SORGAM)^83^. EURAD-IM simulations with an improved representation of the NO_3_· oxidation of biogenic VOCs in the SOA module^84^ gave good agreement with measurements of organic nitrate in Europe^24^. The EURAD-IM was applied with a 9 × 9 km² horizontal resolution and 23 vertical terrain-following layers up to 100 hPa. A spin-up of 5 days for each period was used. Two model sensitivity analyses were performed: (1) the primary emissions of three types of phenolic compounds (phenol, catechol, and cresols) emitted from residential heating were included with an estimated emission ratio of 2.54 ppt ppb^−1^ normalized to CO emitted from residential heating^85^; (2) the SOA yield from the oxidation of phenolic compounds (by both OH· and NO_3_· oxidation) was increased from ~0.02 to ~0.3^56,57^.

## Supplementary information

Supplementary Information

## Data Availability

The data used in this study are available from the Jülich DATA platform (10.26165/JUELICH-DATA/TPPXNL).
