# Supplementary material for: Observational evidence reveals the significance of nocturnal chemistry in seasonal secondary organic aerosol formation
Source: NPJ Clim Atmos Sci. 2024 Sep 6;7(1):207. doi: 10.1038/s41612-024-00747-6 (PMC11379628; doi:10.1038/s41612-024-00747-6)
Supplement: Supplementary file 1 — Supplementary Information [file 41612_2024_747_MOESM1_ESM.pdf]

Supplementary Information for

Observational Evidence Reveals the Significance of Nocturnal Chemistry in Seasonal Secondary Organic Aerosol Formation.

Lu Liu[1,2], Thorsten Hohaus[1]*, Philipp Franke[1], Anne C. Lange[1], Ralf Tillmann[1], Hendrik Fuchs[1,3], Zhaofeng Tan[4], Franz Rohrer[1], Vlassis Karydis[1], Quanfu He[1], Vaishali Vardhan[5], Stefanie Andres[1], Birger Bohn[1], Frank Holland[1], Benjamin Winter[1], Sergej Wedel[1], Anna Novelli[1], Andreas Hofzumahaus[1], Andreas Wahner[1], and Astrid Kiendler-Scharr[1#]

[1] Institute of Energy and Climate Research, IEK-8: Troposphere, Forschungszentrum Jülich GmbH, Jülich, Germany

[2] Laboratory of Atmospheric Chemistry, Paul Scherrer Institute, Villigen, Switzerland

[3] Department of Physics, University of Cologne, Cologne, Germany

[4] State Key Joint Laboratory of Environmental Simulation and Pollution Control, State Environmental Protection Key Laboratory of Atmospheric Ozone Pollution Control, College of Environmental Sciences and Engineering, Peking University, Beijing, China

[5] Environmental Research Centre, University College Cork, Cork, Ireland

[#] deceased

* Thorsten Hohaus

Email: t.hohaus@fz-juelich.de

This PDF file includes:

Supplementary Notes 1 to 3

Supplementary Figures 1 to 25

Supplementary Tables 1 to 6

Supplementary References

Supplementary Note 1. Field Campaign Description

During the JULIAC campaign, intensive measurement periods (4-6 weeks) with comprehensive instrumentations were conducted in each season in 2019: winter (15th Jan. to 10th Feb.); spring (8th Apr. to 5th May); summer (7th Aug. to 1st Sep.); and autumn (28th Oct. to 24th Nov.). Due to the surrounding environment as shown in Supplementary Figure 1, the JULIAC campaign site could be affected by both anthropogenic and biogenic emission sources. The setup of the JULIAC measurement site comprised a 50 m high inlet connected to the atmosphere simulation chamber SAPHIR [1,2]. Details on the JULIAC campaign set up can be found in previous publications [3,4]. In short ambient air was drawn from 50 m height above ground into the chamber via a passivated inlet line (SilcoNert®-coated stainless steel) with an inner diameter of 104 mm. Due to a high sampling flow rate of 660 $m^3\ h^{-1}$ the residence time of the ambient air inside the inlet line was on the order of 4 s which minimized losses and changes in trace gas concentrations and aerosol composition. During nighttime the height of the inlet line ensures that the sampled air is not impacted directly by near-ground emissions or deposition or the forest canopy, focusing on regional atmospheric composition.

Supplementary Note 2. Instrumentation

In this work, the focus is on the physical and chemical characterization of submicron aerosols measured by a high-resolution time-of-flight aerosol mass spectrometer (HR-ToF-AMS, Aerodyne). In this study, two same version of HR-ToF-AMS were utilized in measurement, with one used in winter and spring, and the other one used in summer and autumn. Two HR-ToF-AMS run in mass spectrum (MS) mode with V shape path in the flight chamber, and particle ToF (PToF) mode during the campaign. However, only MS mode data with a time resolution of 2 or 3 minutes, depending on the mode setting, were reported here. To mitigate the impact of instrument changes on the data, collected raw data were analyzed separately for each season. Calibration methods, mainly ion efficiency calibration [5], relative ion efficiency calibration [6], and composition-dependent collection efficiency [7], were also utilized to improve the accuracy and reliability of data.

In addition, gas phase N_2O_5 was measured by a custom-built cavity ring-down spectroscopy (FZJ-CRDS) instrument built following the design in Wagner et al.,2011 [8]. Photolysis frequencies were calculated based on spectral actinic flux densities detected by a spectroradiometer [1,2]. Carbon monoxide (CO), carbon dioxide (CO_2), and methane (CH_4) concentrations were continuously detected by a cavity ring-down instrument (Picarro). Nitrogen dioxide (NO_2) and nitric oxide (NO) were monitored by a chemiluminescence (CL) instrument with a photolytic converter (ECO PHYSICS). O_3 was monitored by UV photometer Comprehensive VOCs concentration were supplied by both proton transfer reaction time of flight mass spectrometer and VOCUS with variation inside.

Supplementary Note 3. PMF analysis

3.1 Organic matrix PMF

In the PMF analysis of this study, unconstrained PMF runs were firstly performed for measured aerosol organics by the HR-ToF-AMS instrument for each season by rotational techniques, Seed and Fpeak, with a scan range of 2-10 factors to explore the appropriate number of source factors. Then, constrained PMF analysis (mainly a-value approach) was executed with determined proper factor numbers and factor profiles (normally a primary factor, like HOA) from previous studies or this study. For the PMF main setting, the signal-to-noise ratio (S/N) threshold for the unexplained variation was set to 2. S/N function and cell-wise data type were applied to downweigh the error signal with S/N lower than 1 for each cell separately [9]. CO_2^+ related ions (O^+, HO^+, H_2O^+, and CO^+) were downweighed with a factor of 2.24 in case of the excessive weighting of CO_2^+. Completed seasonal overview of PMF analysis for measurements of organic aerosol, including factor spectrum and diurnal pattern, were displayed in Supplementary Figure 10-13. For biomass burning sources, the emission types were different among seasons. The BBOA resolved by PMF during spring was shown to be related to regional transport of wildfire plumes based on NASA satellite fire map coupled with back-trajectory analysis (calculated by PC-based Hybrid Single-Particle Lagrangian Integrated Trajectory model, version 5.0.0). Contrarily, during winter and autumn, BBOA resolved by PMF analysis was found to be not correlated to episodic events which relates to possibly more general human activities, like residential heating.

3.2 Organic and Nitrate matrix PMF

The PMF analysis for the measurements of aerosol organics and nitrate was also utilized in this study. The constrained PMF analysis by a-value methods (exact a-value 0.1-0.2) was conducted for the matrices of nitrate and organics for four seasons using prior factors from the optimal results of PMF analysis for organics only. It should be noticed that fragment NO^+ and NO_2^+ position was not constrained by prior factors. The determination of source factors in the nitrate+organics PMF analysis mainly relies on the correlation of factor spectra and time series with the results of the PMF analysis of organics, as shown in Supplementary Table 5. The overview of the optimal nitrate+organic PMF run during the JULIAC campaign is displayed in Supplementary Figure 14-17. Overall, in the results of nitrate+organic PMF analysis, nitrate fragments were barely attributed to HOA, BBOA, LO-OOA, and MO-OOA factors. Therefore, they are properly determined, since their overall time series, diurnal pattern, and profile characteristics (such as elemental ratio) were almost the same as those of the organic PMF results. For nocturnal oxidation and regional transport sources, the spectra of their source factors determined by nitrate+organic PMF show obvious changes due to nitrate fragments distribution, but the time series of their source factors still show high similarity to corresponding source factors in the results of the PMF analysis for organics. In addition, one more OA source with rich nitrate components (called NO_3-OA) was determined during nitrate+organic PMF analysis. That NO_3-OA factor showed predominantly aerosol inorganic nitrate variation since it has a similar NO_2^+/NO^+ ratio compared to pure NH_4NO_3 detected by AMS during calibration. A NO_3-OA factor was resolved during summer and autumn, while during winter and spring, it was mixed with the regional transport factor. Therefore, in the latter two seasons, we utilize RT-NO_3-OA to denote a possible regional transport of ammonium nitrate rich plumes (correspond to the regional transport OA in Figure 1). In addition, the robustness of the results of the PMF analysis for organic+nitrate was inspected by 200 PMF bootstrapped runs conducted for each season by constraining results, using random a-values with a step of 0.1 and ranging from 0 to 0.5. The average and spread of the solution in the bootstrap analysis are displayed in Supplementary Figure 18

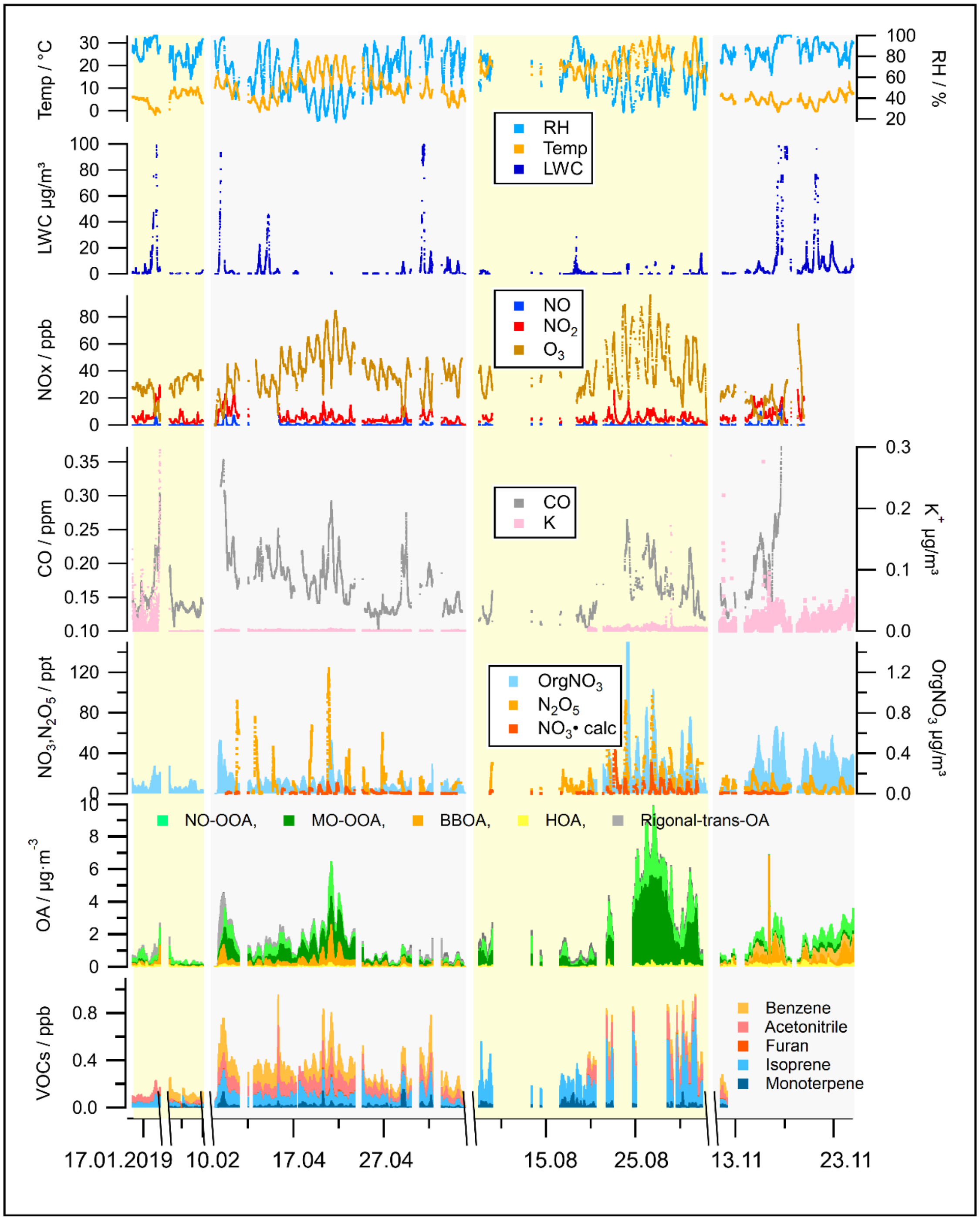

Supplementary Figure 1. Overview of variations in concentration of organic aerosol sources resolved by PMF analysis, aerosol LWC (ALWC), trace gases (VOCs, CO, O_3, NO_x, N_2O_5, NO_3 radicals), and meteorological parameters (temperature and RH) during the whole JULIAC campaign.

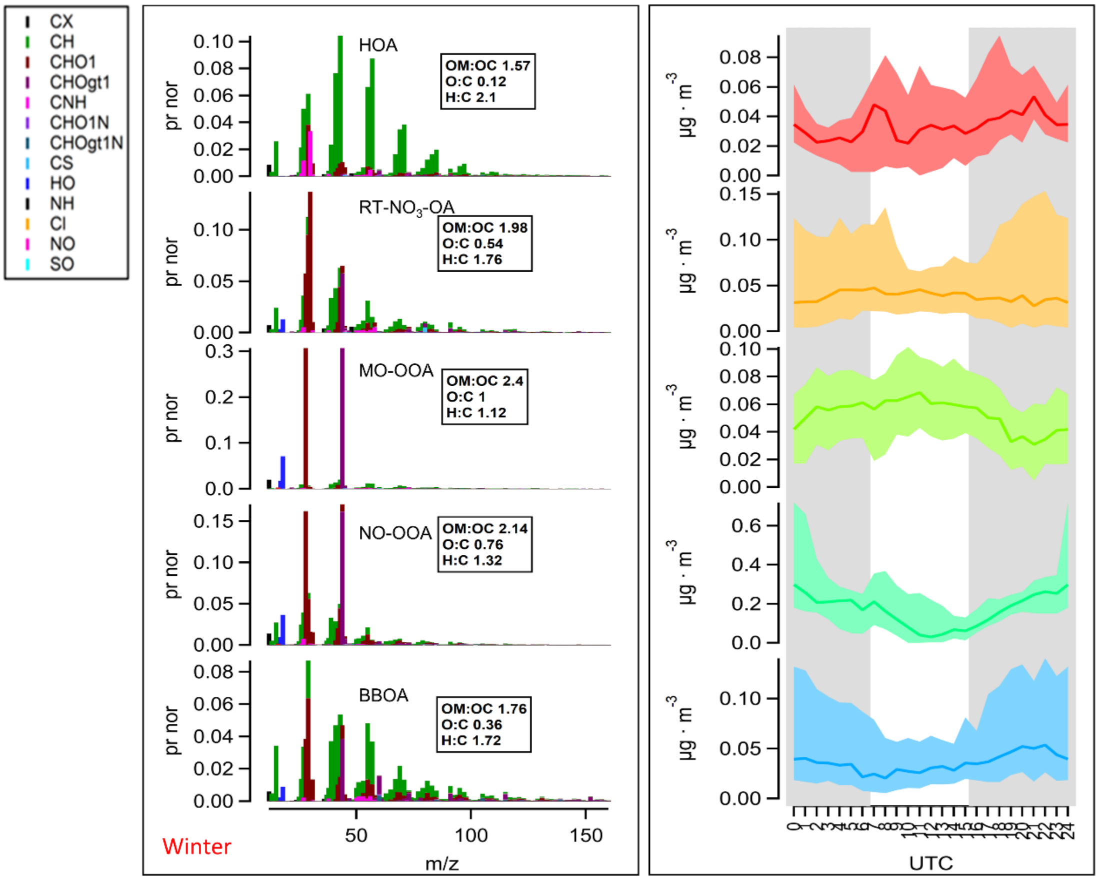

Supplementary Figure 2. The results of the source apportionment resolved by PMF analysis of organic aerosol for the winter of 2019. From left to right, the graph shows the variation of contribution to OA for each source factor, i.e., the high-resolution spectra of source factors colored by the family group of ions, and the diurnal pattern of the median (solid line) and interquartile range (IQR, color block) of the contribution of the corresponding source factor. The elemental ratio (OM:OC, O:C, H:C) of all OA factors is shown in the graph.

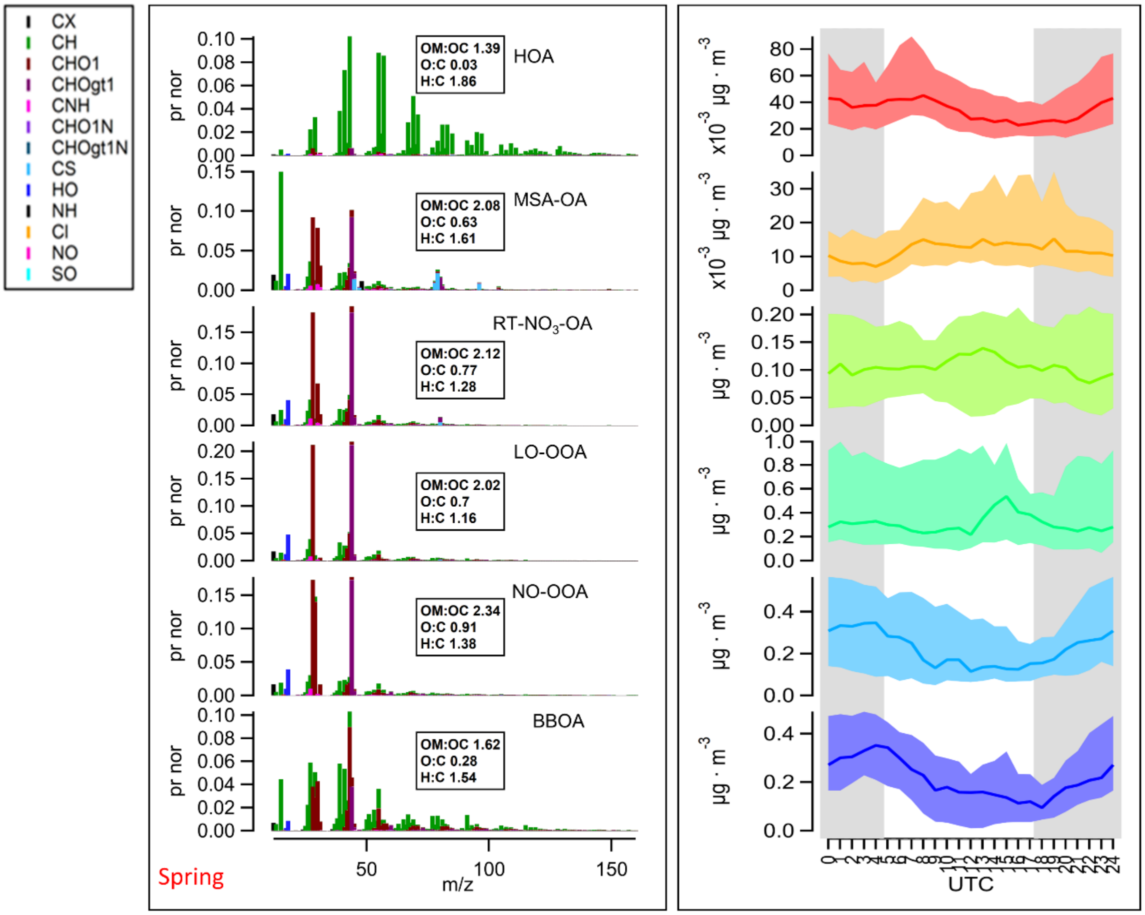

Supplementary Figure 3. The results overview of source apportionment resolved by PMF analysis of organic aerosol for the spring of 2019. From left to right, the graph shows the variation of contribution to OA for each of source factor, i.e. high-resolution spectra of source factors colored by the family group of ions, and the diurnal pattern of the median (solid line) and interquartile range (IQR, color block) of the contribution of the corresponding source factor. The elemental ratio (OM:OC, O:C, H:C) of all OA factors is shown in the graph.

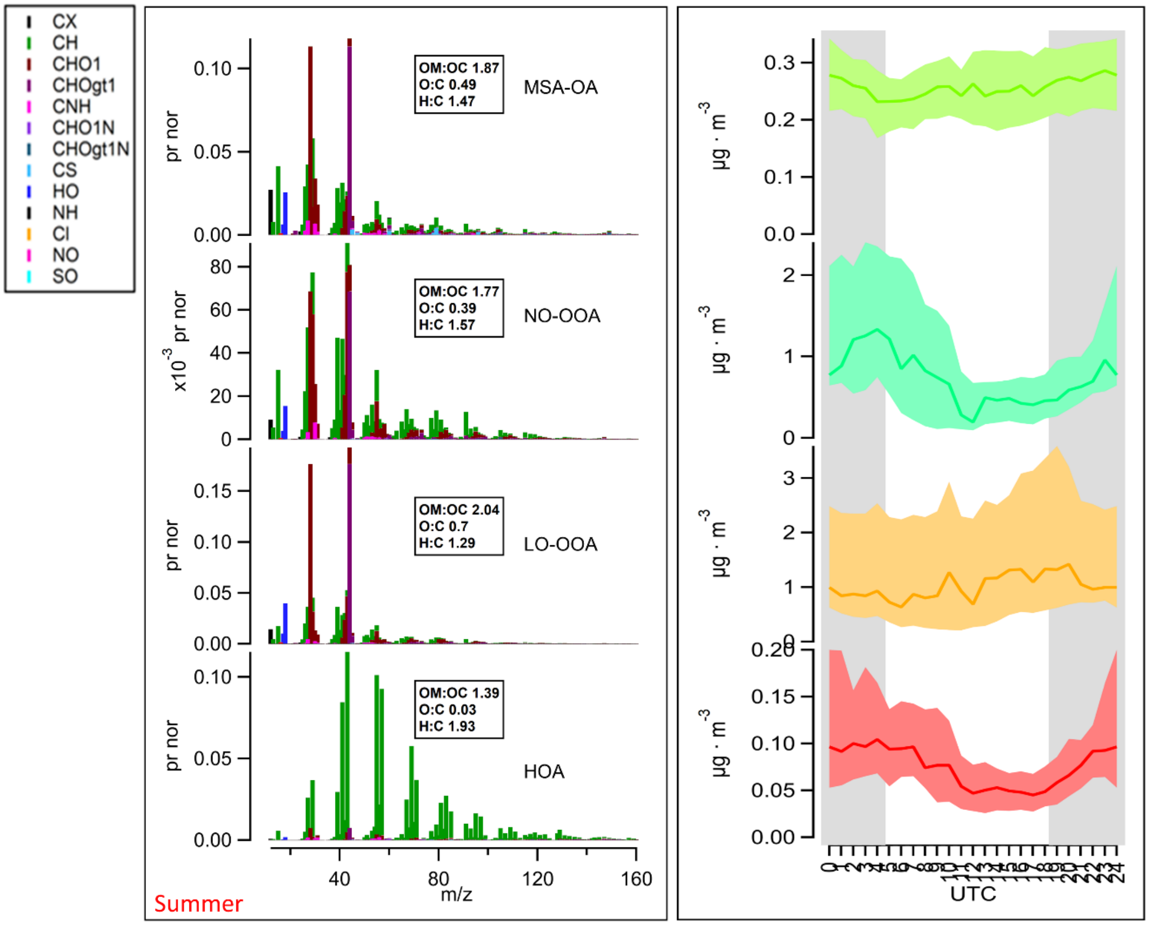

Supplementary Figure 4. The results overview of source apportionment resolved by PMF analysis of organic aerosol for the summer of 2019. From left to right, the graph shows the variation of contribution to OA for each source factor, i.e. the high-resolution spectra of source factors colored by the family group of ions, and the diurnal pattern of the median (solid line) and interquartile range (IQR, color block) of the contribution of the corresponding source factor. The elemental ratio (OM:OC, O:C, H:C) of all OA factors is shown in the graph.

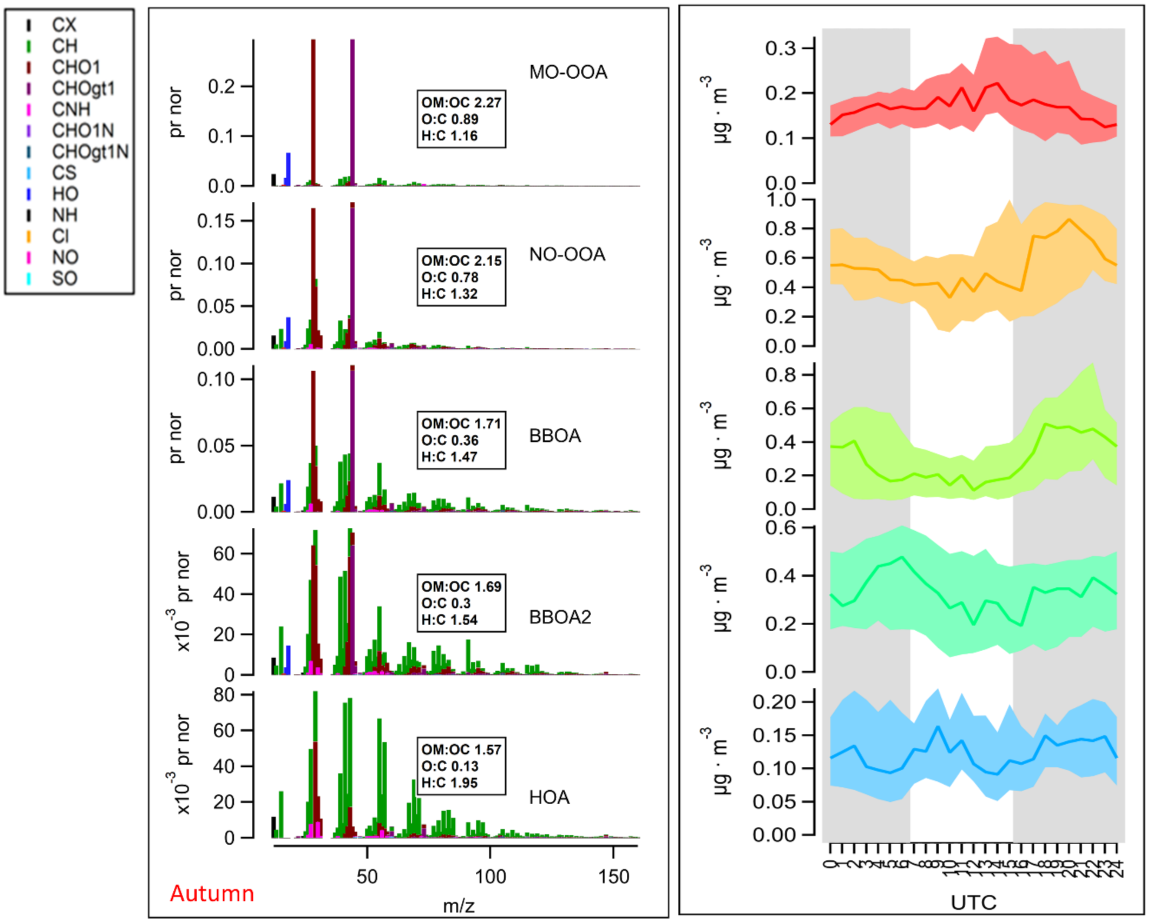

Supplementary Figure 5. The results overview of source apportionment resolved by PMF analysis of organic aerosol for the autumn of 2019. From left to right, the graph shows the variation of contribution to OA for each source factor, i.e. the high-resolution spectra of source factors colored by the family group of ions, and the diurnal pattern of the median (solid line) and interquartile range (IQR, color block) of the contribution of the corresponding source factor. The elemental ratio (OM:OC, O:C, H:C) of all OA factors is shown in the graph.

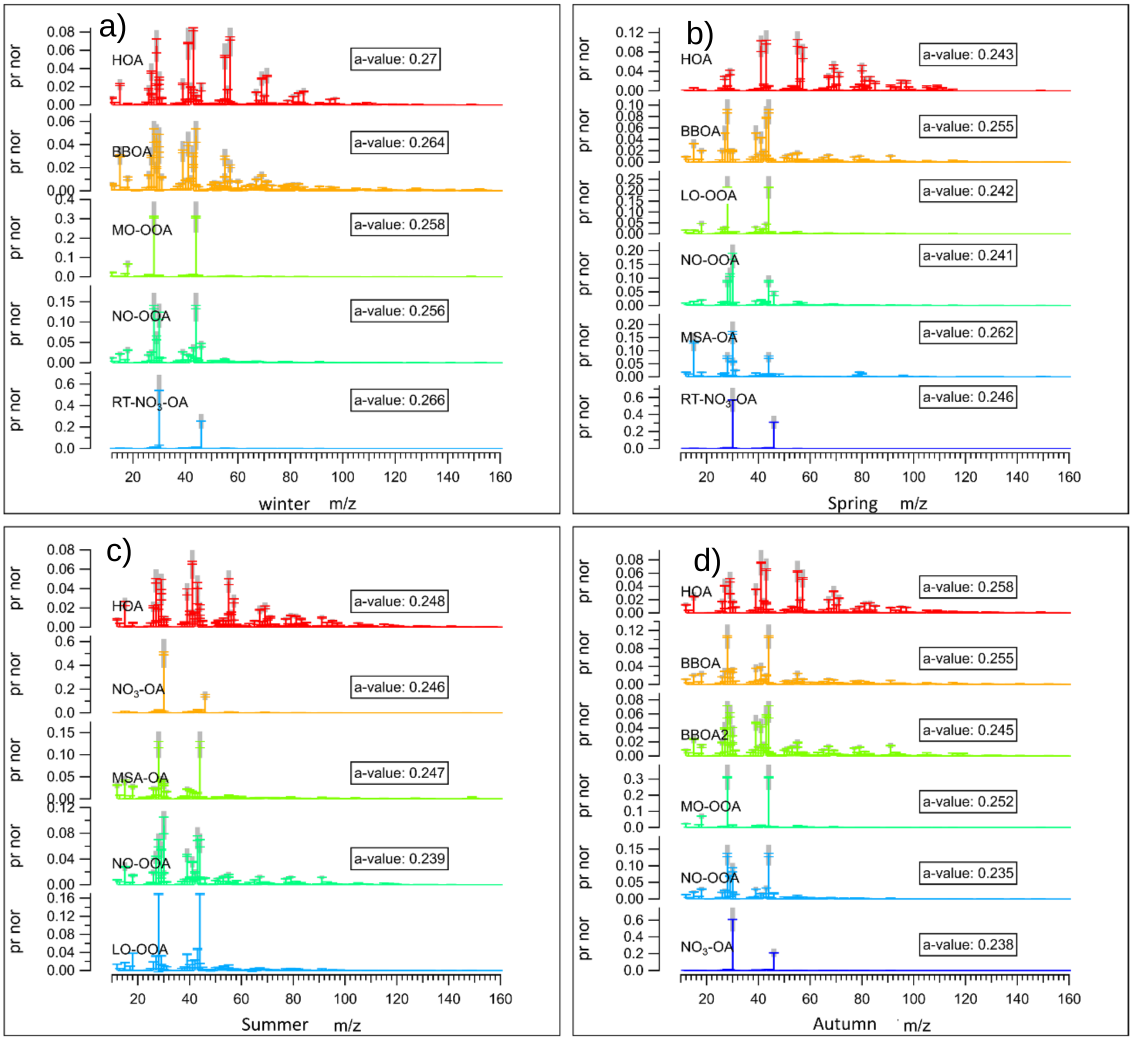

Supplementary Figure 6. Averaged factor profiles (m/z 10-160) of 200 bootstrap runs of each seasonal PMF analysis for aerosol nitrate and organics during the JULIAC campaign, a) winter, b) spring, c) summer and d) autumn, with random a-value methods. The standard deviation of the averaged bootstrapped run is displayed as error bars. The shaded areas represent the constraints of random a-value methods, and the averaged a-value for corresponding factors is also displayed in the graph.

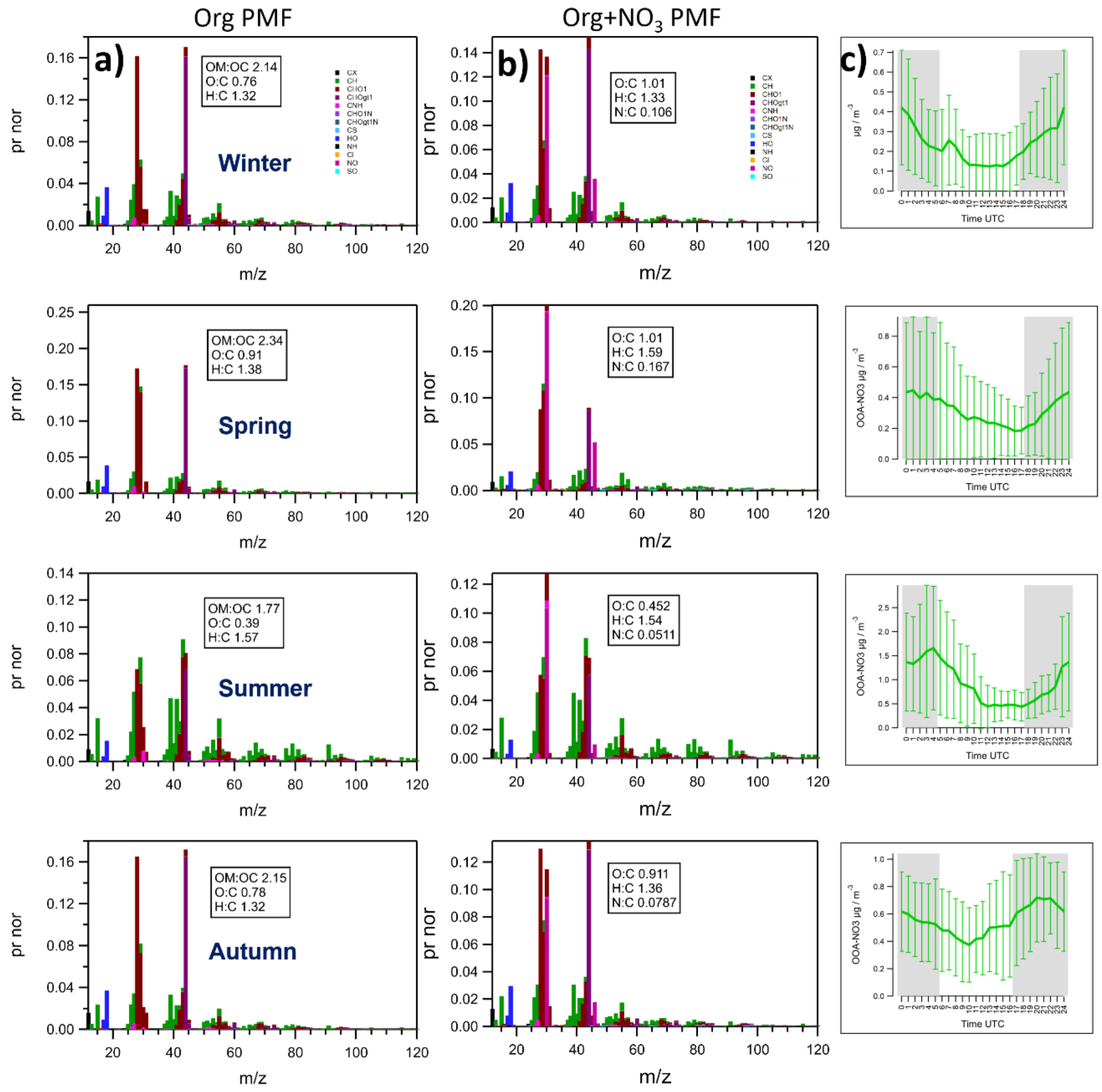

Supplementary Figure 7. a) The high-resolution mass spectra of NO-OOA resolved by PMF analysis of aerosol organics for the four seasons of the JULIAC campaign, colored by family groups of ions. The y-axis presents the intensity fraction of the ion signal, while the x-axis shows the mass-to-charge ratio (m/z) of ions ranging from 12 to 120. The elemental ratio (OM:OC, O:C, H:C) of NO-OOA has been displayed. b) The high-resolution mass spectra of NO-OOA resolved by the PMF analysis of measurements of aerosol nitrate and organics during the whole JULIAC campaign, colored by family groups of ions. The intensity ratio of nitrate ions, NO_2^+/NO^+, and the elemental ratio (O:C, H:C and N:C) of the NO-OOA are displayed for each season. The y-axis

presents the ion signal intensity fraction, while the x-axis is the mass-to-charge ratio (m/z) of ions ranging from 12 to 120. c) The averaged diurnal variation of NO-OOA concentrations with standard deviation as error bars during the whole JULIAC campaign. The grey background in the graph denotes night-time hours, while the white background marks daytime hours characterized by measurements of corresponding photolysis frequency.

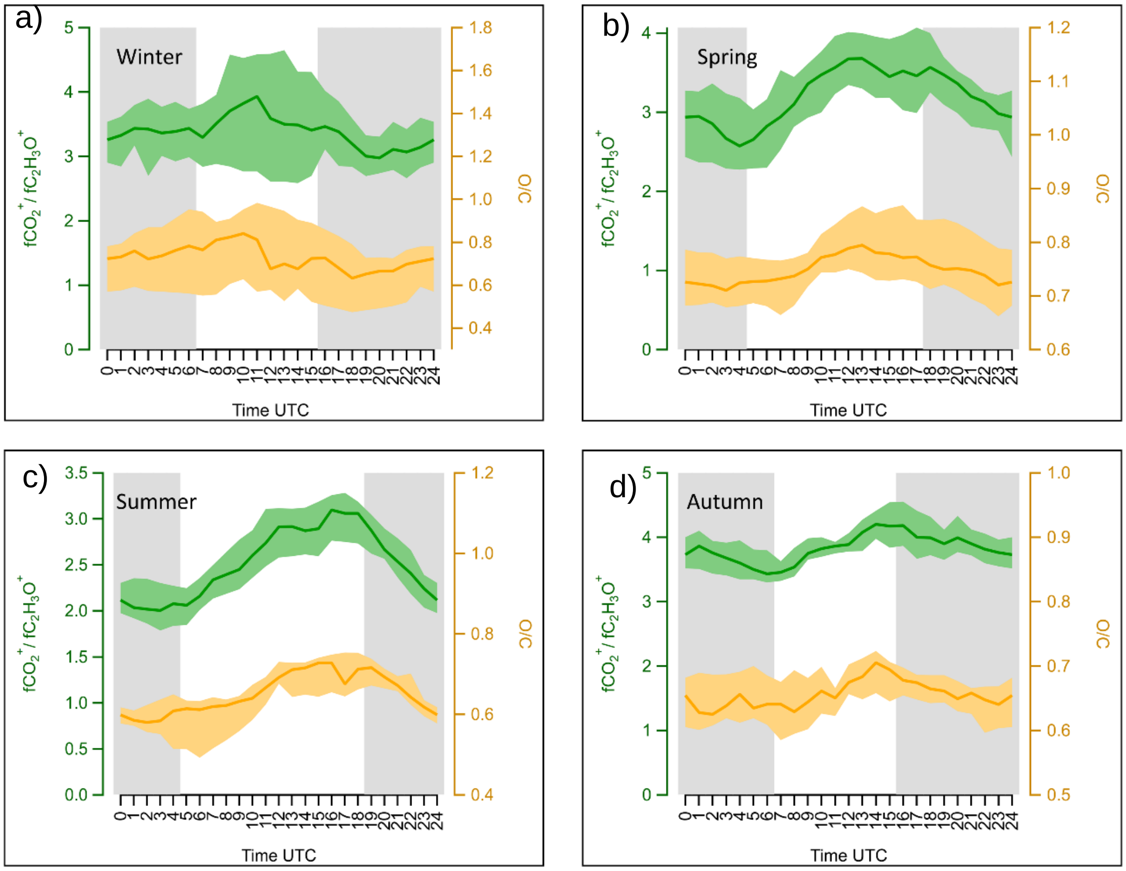

Supplementary Figure 8. Diurnal variations of the median and interquartile range (IQR) of elemental ratio O:C and fragment ratio $fCO_2^+/fC_2H_3O^+$ of organic aerosol for all four seasons, a) winter, b) spring, c) summer and d) autumn, during the JULIAC campaign. Solid lines correspond to median variations and color regions represent the IQR. Grey background indicates night-time and white background marks daytime determined by the average of photolysis frequency data measured for different seasons. Note that the scale of the y-axis is different among the four seasons.

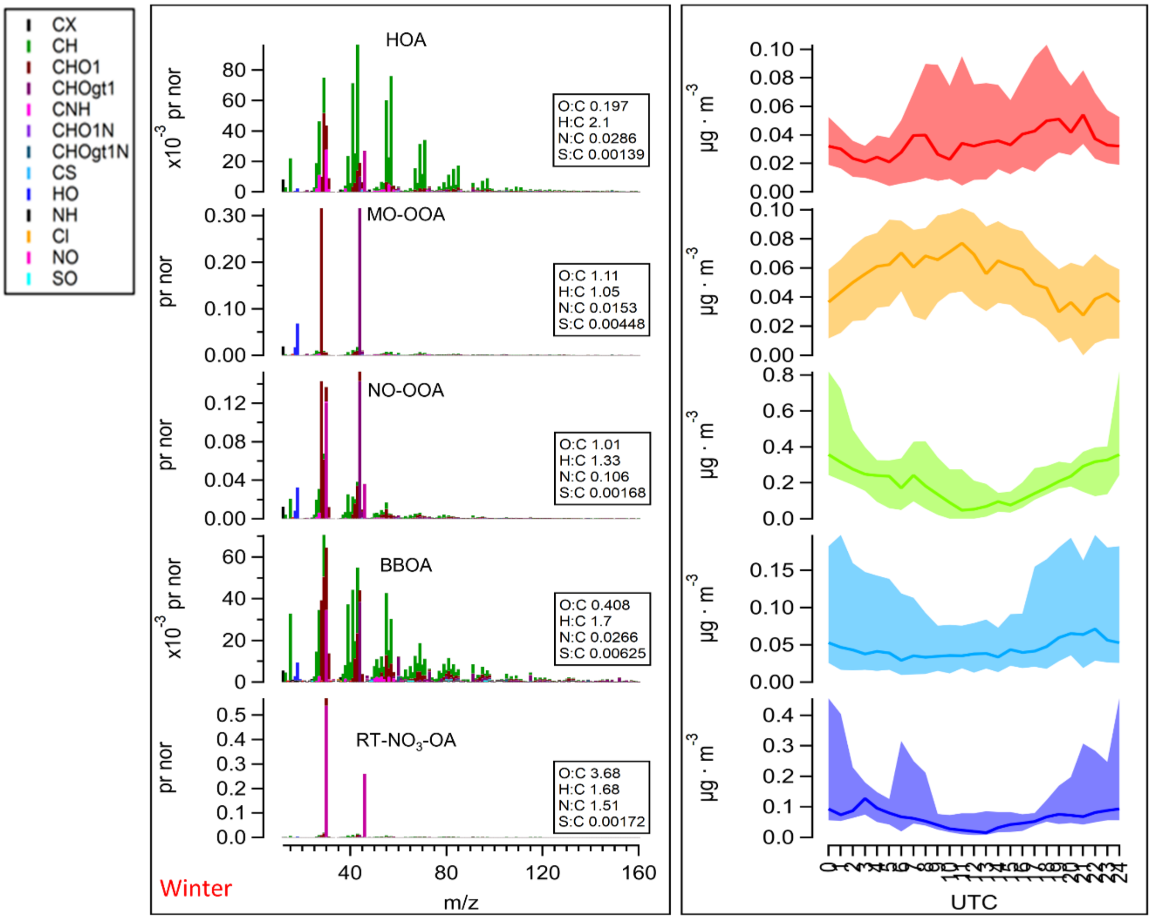

Supplementary Figure 9. The results overview of source apportionment resolved by PMF analysis of aerosol nitrate and organics for the winter of 2019. From left to right, the graph shows the variation of contribution to OA for each source factor, i.e. the high-resolution spectra of source factors colored by the family group of ions, and the diurnal pattern of the median (solid line) and interquartile range (IQR, color block) of the contribution of the corresponding source factor. The elemental ratio (OM:OC, O:C, H:C) of all OA factors is shown in the graph.

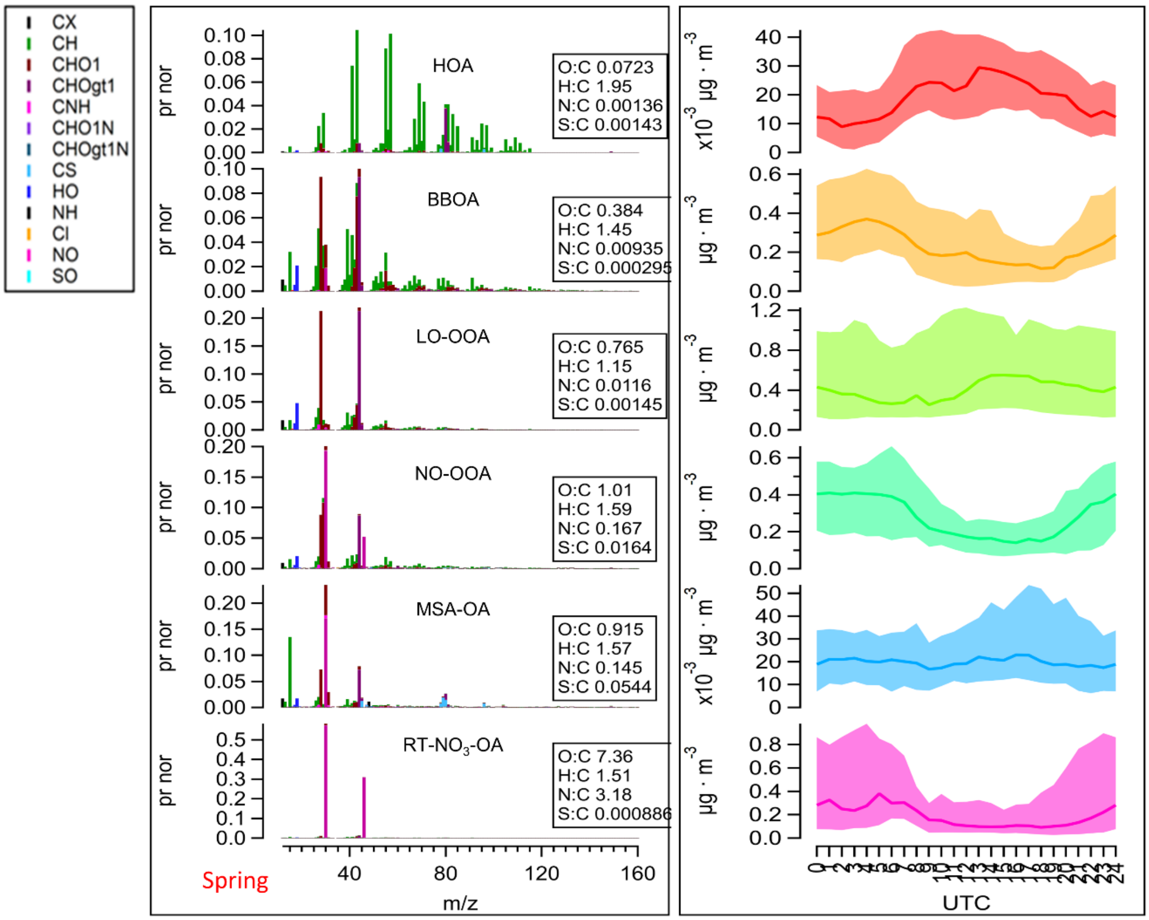

Supplementary Figure 10. The results overview of source apportionment resolved by PMF analysis of aerosol nitrate and organics for the spring of 2019. From left to right, the graph shows the variation of contribution to OA for each source factor, i.e. the high-resolution spectra of source factors colored by the family group of ions, and the diurnal pattern of the median (solid line) and interquartile range (IQR, color block) of the contribution of the corresponding source factor. The elemental ratio (OM:OC, O:C, H:C) of all OA factors is shown in the graph.

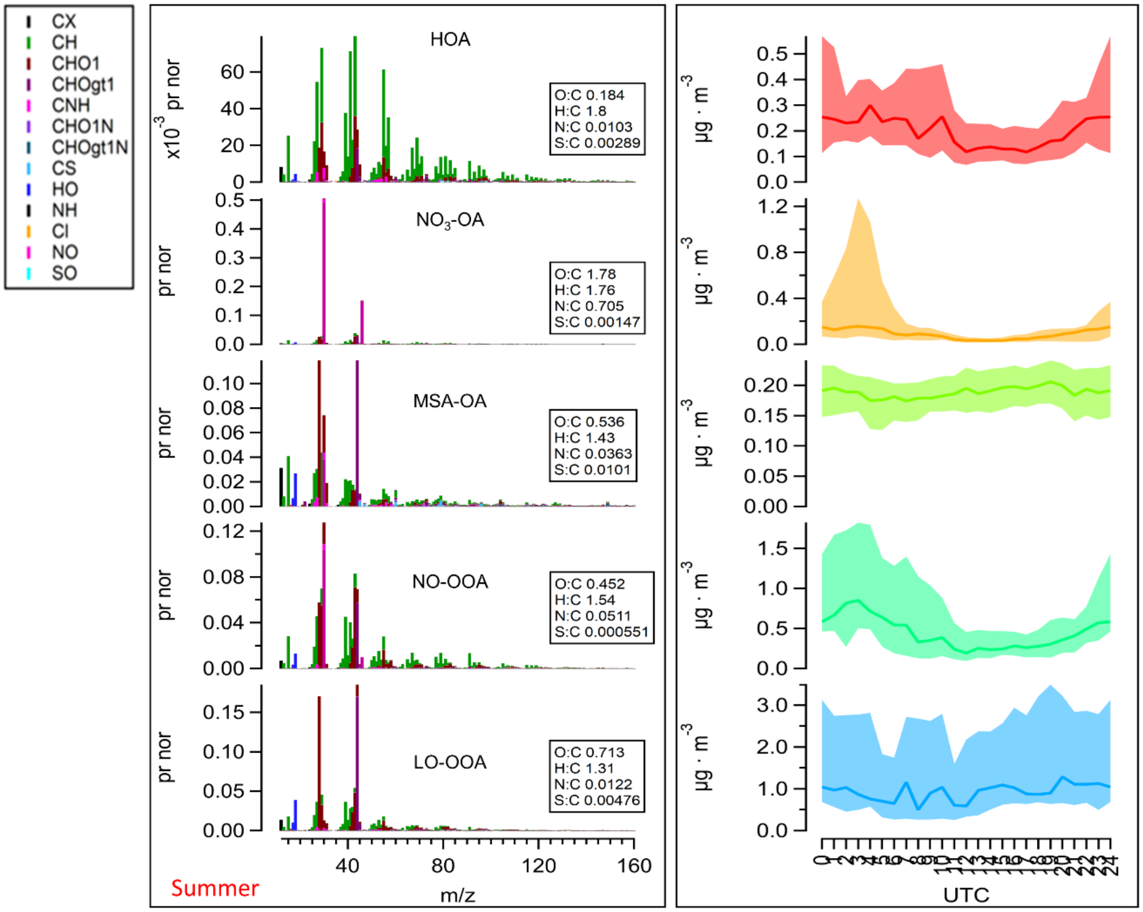

Supplementary Figure 11. The results overview of source apportionment resolved by PMF analysis of aerosol nitrate and organics for the summer of 2019. From left to right, the graph shows the variation of contribution to OA for each source factor, i.e. the high-resolution spectra of source factors colored by the family group of ions, and the diurnal pattern of the median (solid line) and interquartile range (IQR, color block) of the contribution of the corresponding source factor. The elemental ratio (OM:OC, O:C, H:C) of all OA factors is shown in the graph.

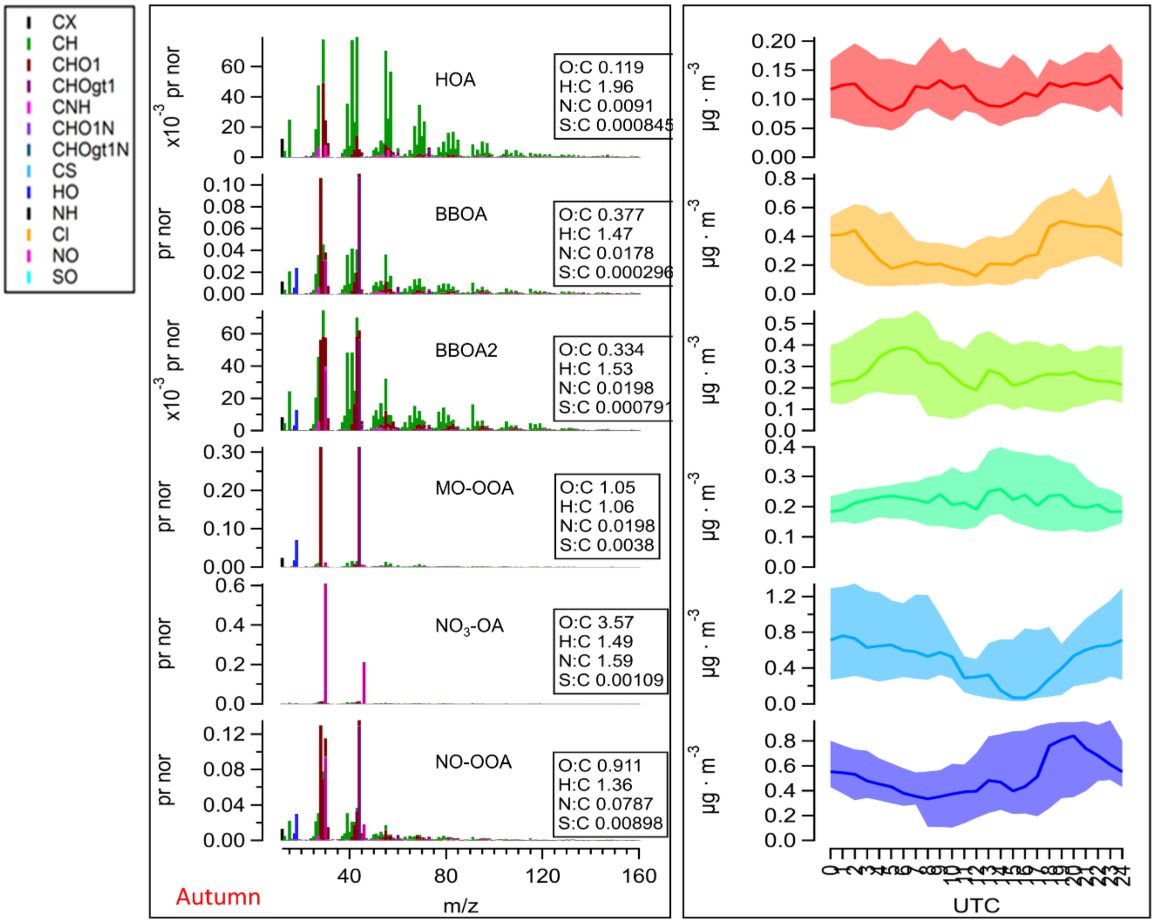

Supplementary Figure 12. The results overview of source apportionment resolved by PMF analysis of aerosol nitrate and organics for the autumn of 2019. From left to right, the graph shows the variation of contribution to OA for each source factor, i.e. the high-resolution spectra of source factors colored by the family group of ions, and the diurnal pattern of the median (solid line) and interquartile range (IQR, color block) of the contribution of the corresponding source factor. The elemental ratio (OM:OC, O:C, H:C) of all OA factors is shown in the graph.

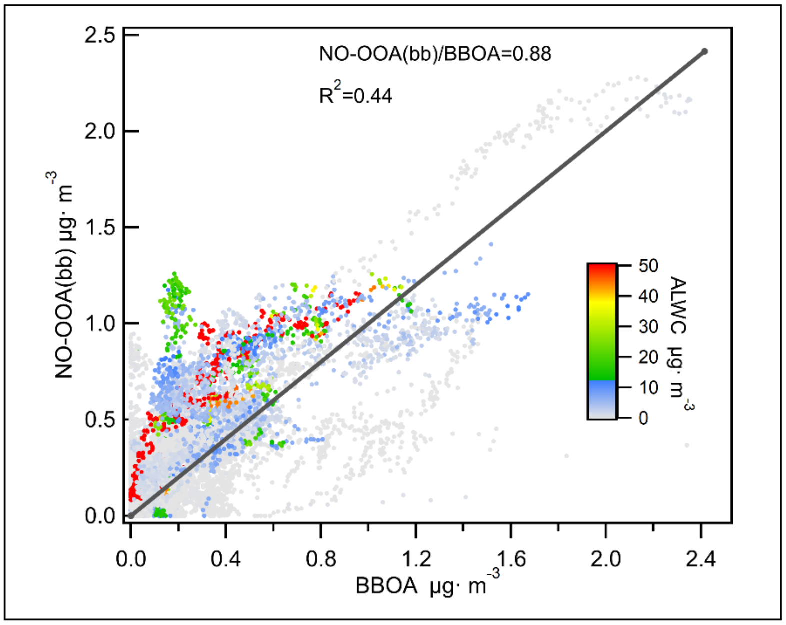

Supplementary Figure 13. Correlation analysis between the concentration of primary BBOA and NO-OOA(bb) resolved by the PMF analysis of OA measurements during winter, spring, and autumn. The color of the dots indicates different levels of aerosol liquid water content (ALWC) calculated by ISORROPIA-II. The linear fit is applied for all data points resulting in a slope of 0.88 and a correlation coefficient of R^2=0.44.

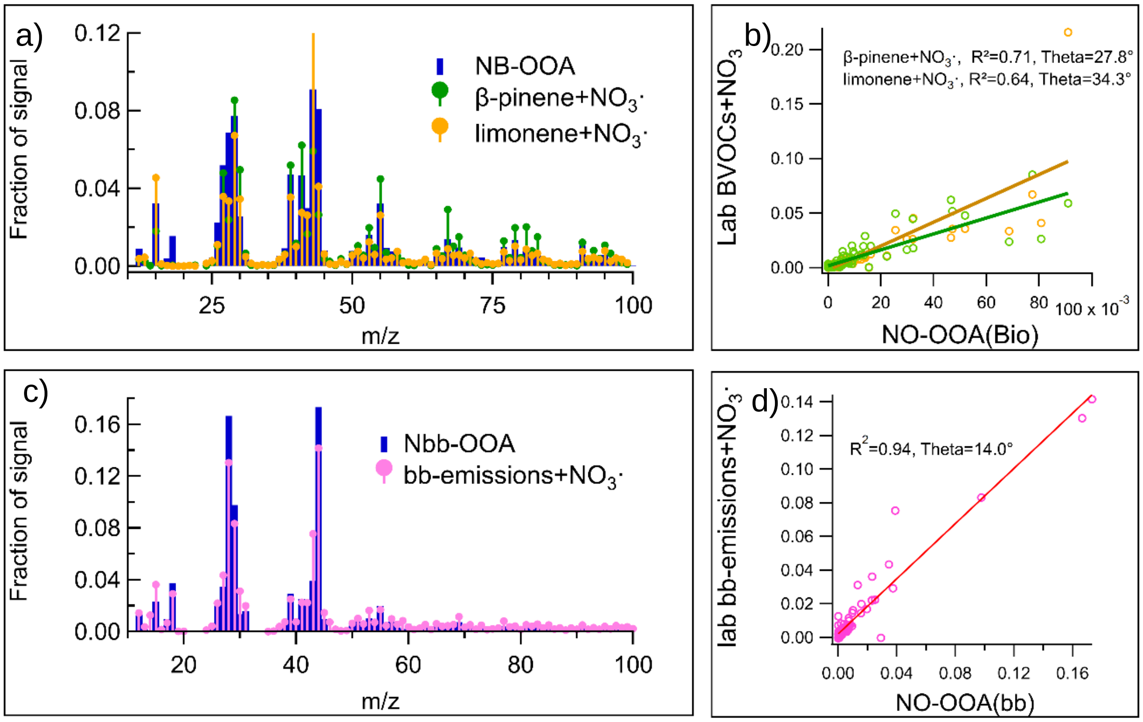

Supplementary Figure 14: The averaged mass spectra of c) NO-OOA(bb) and a) NO-OOA(Bio) were compared with the mass spectra observed in chamber experiments that investigated the production of organic aerosol by the b) NO_3-initiated oxidation of monoterpenes and d) biomass-burning emissions, respectively. The correlation coefficient R^2 and theta angle [10] between the spectra shows their similarity.

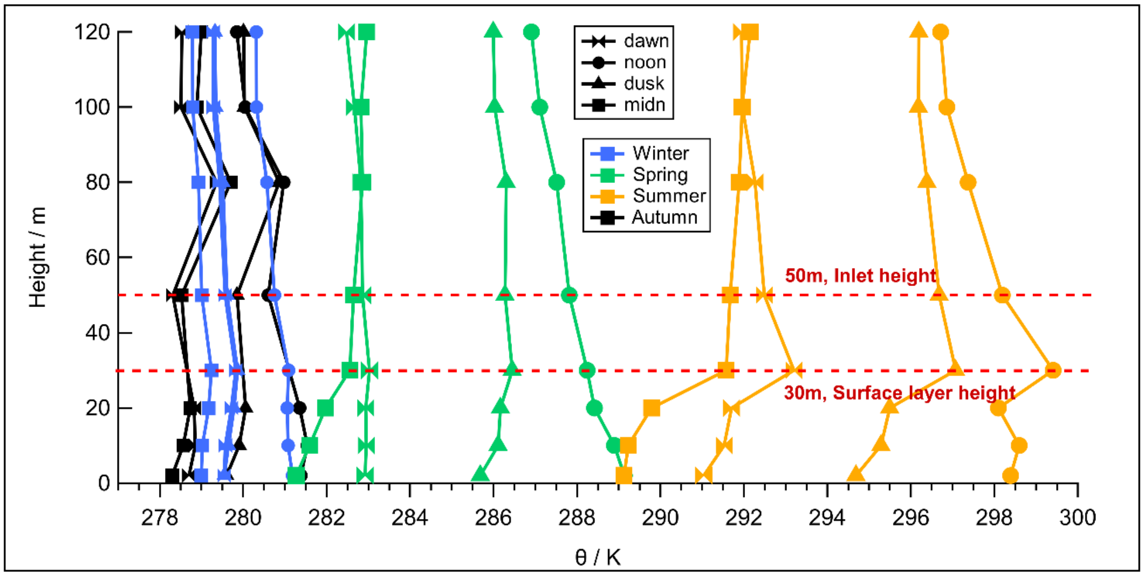

Supplementary Figure 15. The averaged potential temperature profiles for the four seasons during the JULIAC campaign based on gradient measurements of ambient temperature at heights 2 m, 10 m, 20 m, 30 m, 50 m, 80 m, 100 m, and 120 m at the campaign location. Daily variations of potential temperature are sorted into four main periods and are averaged: midnight (22:00-3:00 UTC); dawn (4:00-9:00 UTC); noon (10:00-15:00 UTC) and dusk (16:00-21:00 UTC).

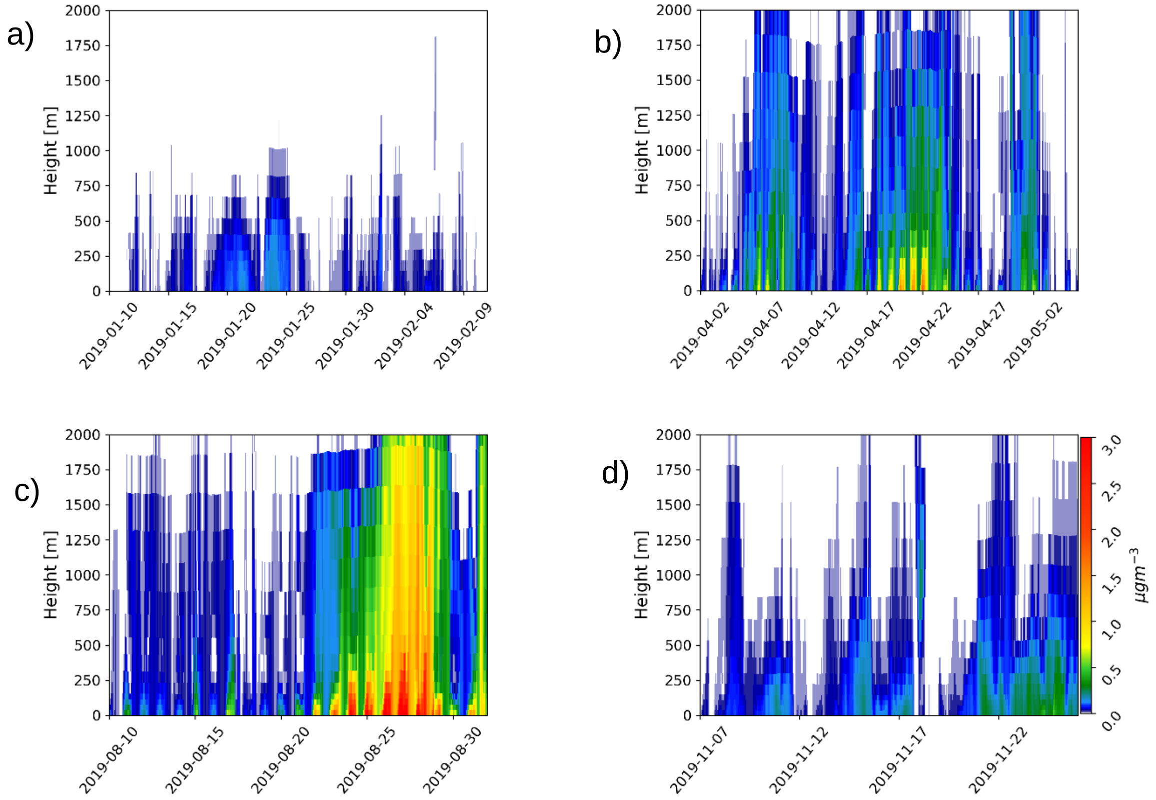

Supplementary Figure 16. The vertical distribution of NO_3-SOA concentration modeled by EURAD-IM for different seasons, a) winter, b) spring, c) summer and d) autumn, in the higher most 2000 m of the troposphere. The NO_3-SOA concentrations show a clear nocturnal enhancement at the ground, which illustrates the significant NO_3-SOA formation from nocturnal oxidation instead of aerosol accumulation in the lowermost troposphere.

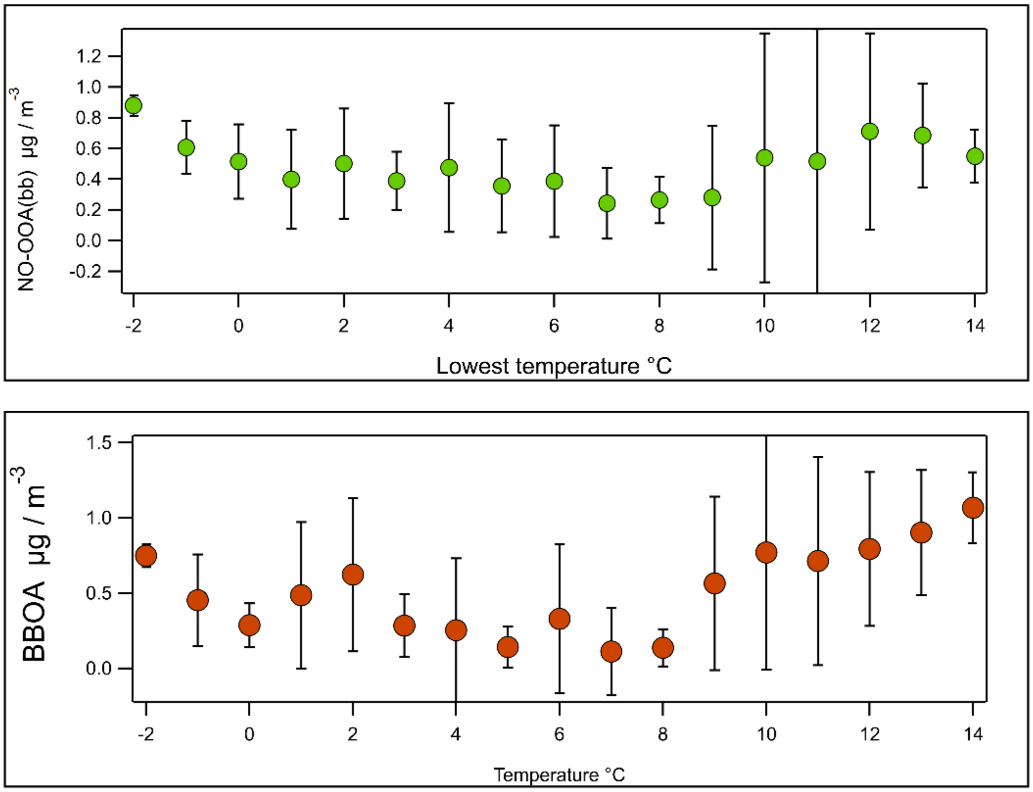

Supplementary Figure 17: The variation of averaged mass concentration and standard derivation of NO-OOA (bb) and BBOA for the whole year excluding summer as a function of the minimum nocturnal temperature. Data is restricted to the period ±2 hours of the minimum temperature.

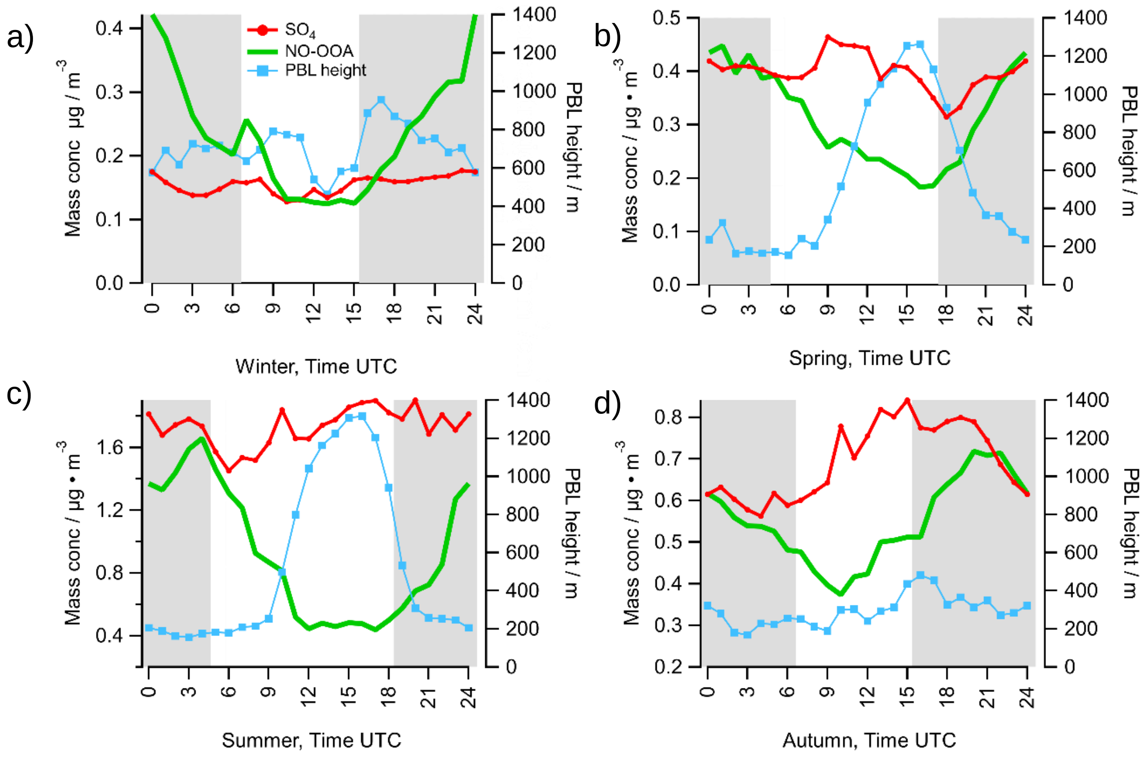

Supplementary Figure 18. Diurnal variation of the seasonally averaged mass concentration of aerosol bulk sulfate (SO_4) and the NO-OOA factor from ground observation, as well as the PBL height simulated by EURAD-IM for all seasons, a) winter, b) spring, c) summer and d) autumn, during the JULIAC campaign.

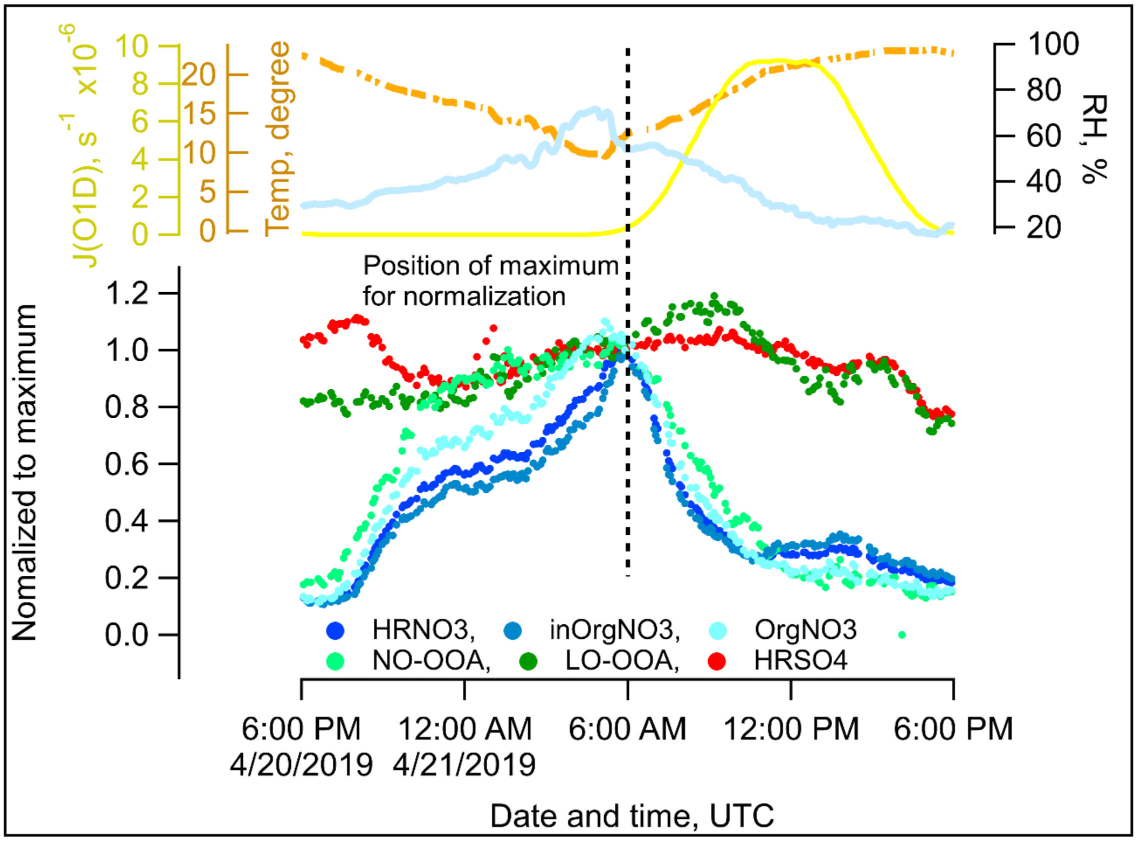

Supplementary Figure 19. The comparison of the variation in concentration of total aerosol nitrate ($HRNO_3$) and sulfate ($HRSO_4$) measured by HR-ToF-AMS, and calculated organic nitrate ($OrgNO_3$), inorganic nitrate ($inOrgNO_3$), as well as the concentrations of NO-OOA(bb) and LO-OOA resolved by PMF analysis during spring. Concurrent temperature (Temp), relative humidity (RH) and photolysis frequency (JO1D) are also shown.

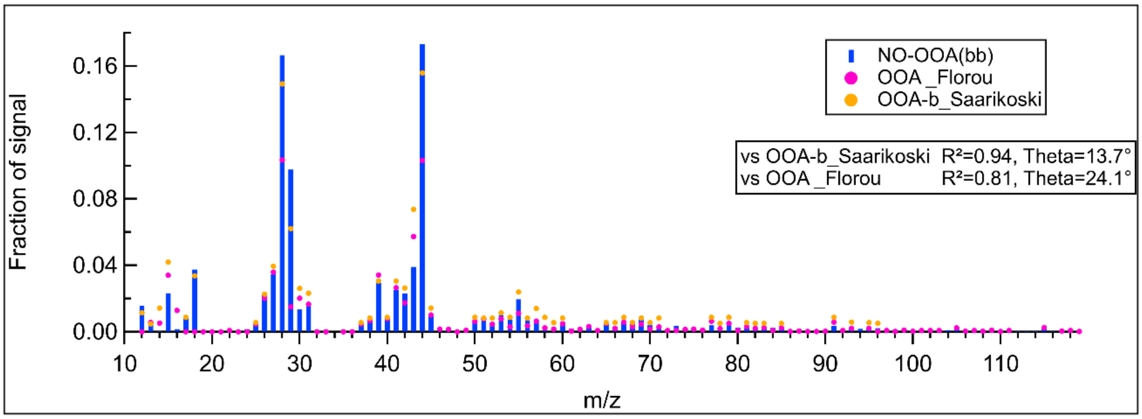

Supplementary Figure 20. The comparison of mass spectra between NO-OOA(bb) (average of winter, spring, and autumn) and ambient uncertain OOA from previous winter measurements [11,12] in unit mass resolution. The similarity of mass spectra of these OOA was shown with correlation coefficient R^2 and theta angle. The y-axis presents the intensity fraction of the ion signal while the x-axis represents m/z (mass-to-charge ratio) ranging from 10 to 120.

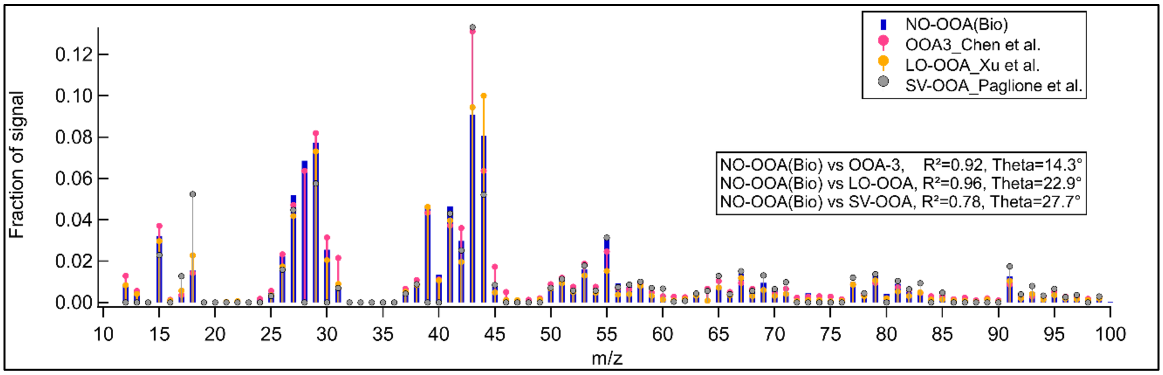

Supplementary Figure 21. The comparison of mass spectra between the NO-OOA(Bio) resolved during summer in this study and ambient biogenic derived OOA factors [13-15] likely related to nocturnal oxidation in unit mass resolution. Only m/z ≤ 100 of spectra are considered because the contribution of ions at m/z > 100 is negligible. Linear correlation analysis between the spectra of NO-OOA(Bio) and biogenic-derived OOA factors of ambient experiment in unit mass resolution has been made and correlation coefficients are also displayed in the graph. The correlation coefficient R^2 and theta angle are marked in the graph to illustrate the spectrum similarity.

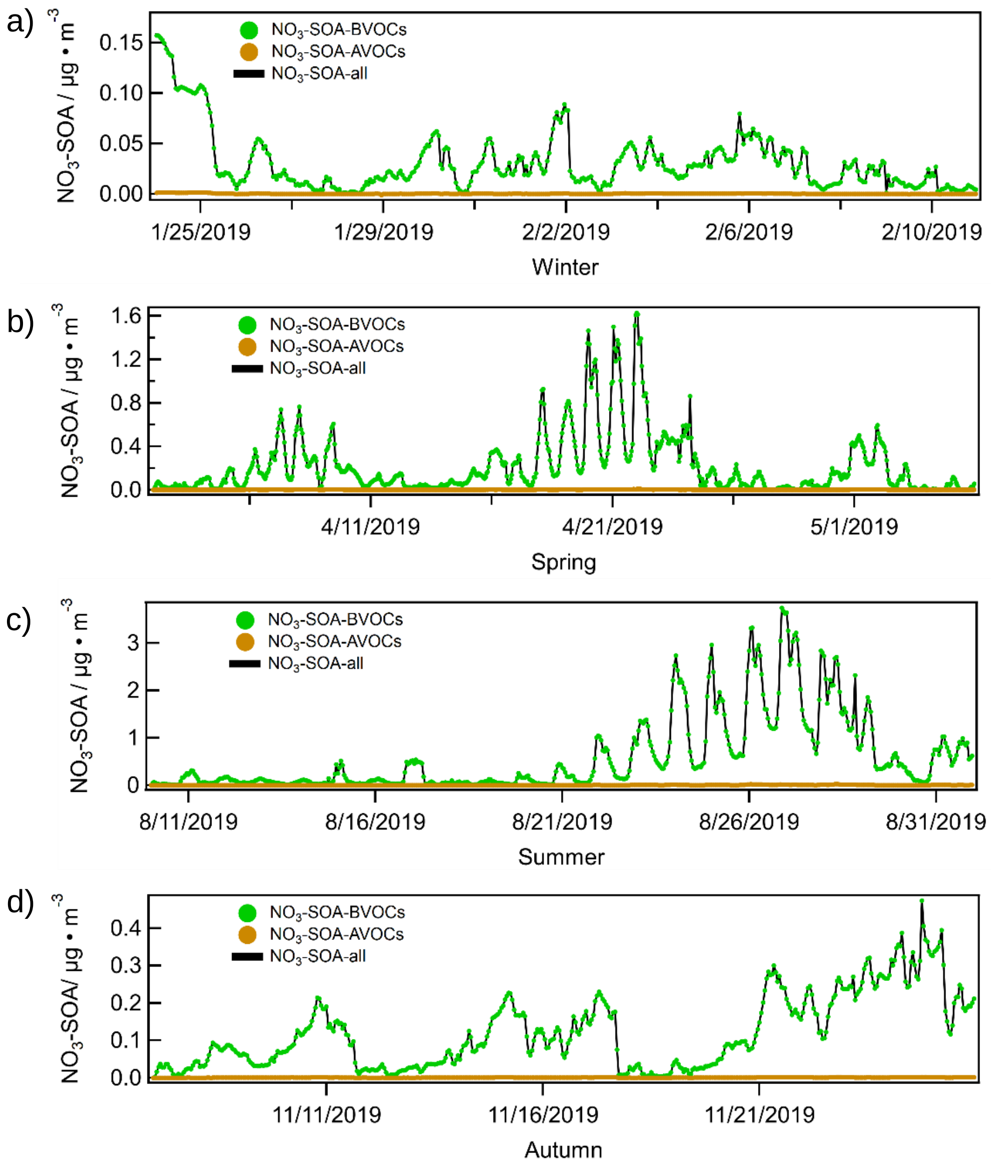

Supplementary Figure 22. NO_3-SOA produced from biogenic VOCs (isoprene+monoterpene; labeled as NO_3-SOA-BVOCs) and anthropogenic VOCs (NO_3-SOA-AVOCs) are separately determined by the EURAD-IM. Here the comparison of modeled NO_3-SOA-BVOCs, NO_3-SOA-AVOCs, and NO_3-SOA-all for the four seasons, a) winter, b) spring, c) summer and d) autumn, during the JULIAC campaign shows that almost all NO_3-SOA concentration predicted by EURAD-IM are of biogenic origin.

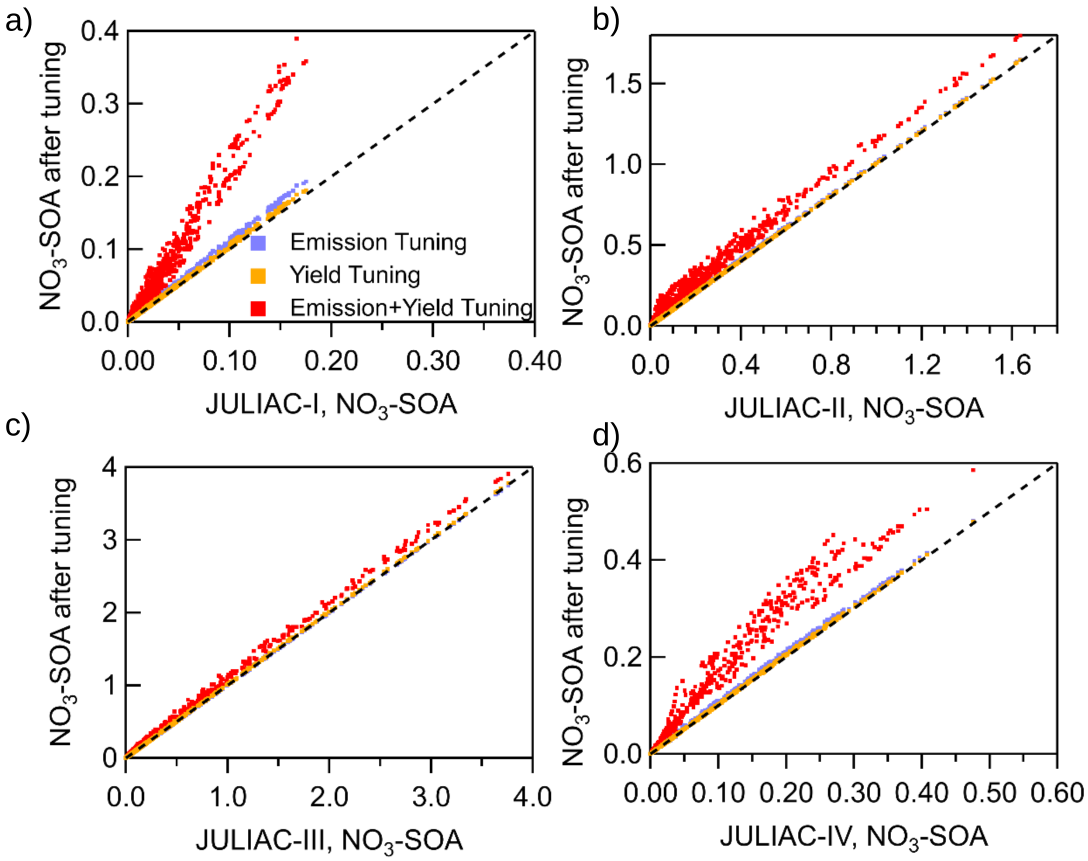

Supplementary Figure 23. The comparison of NO_3-SOA concentrations calculated by EURAD-IM in different seasons, a) winter, b) spring, c) summer and d) autumn, during the JULIAC campaign with that calculated by EURAD-IM after tuning just primary phenolics emissions, just the SOA yield of phenolics oxidation and emission+yield tuning, respectively. The black dashed line illustrates the 1:1 ratio.

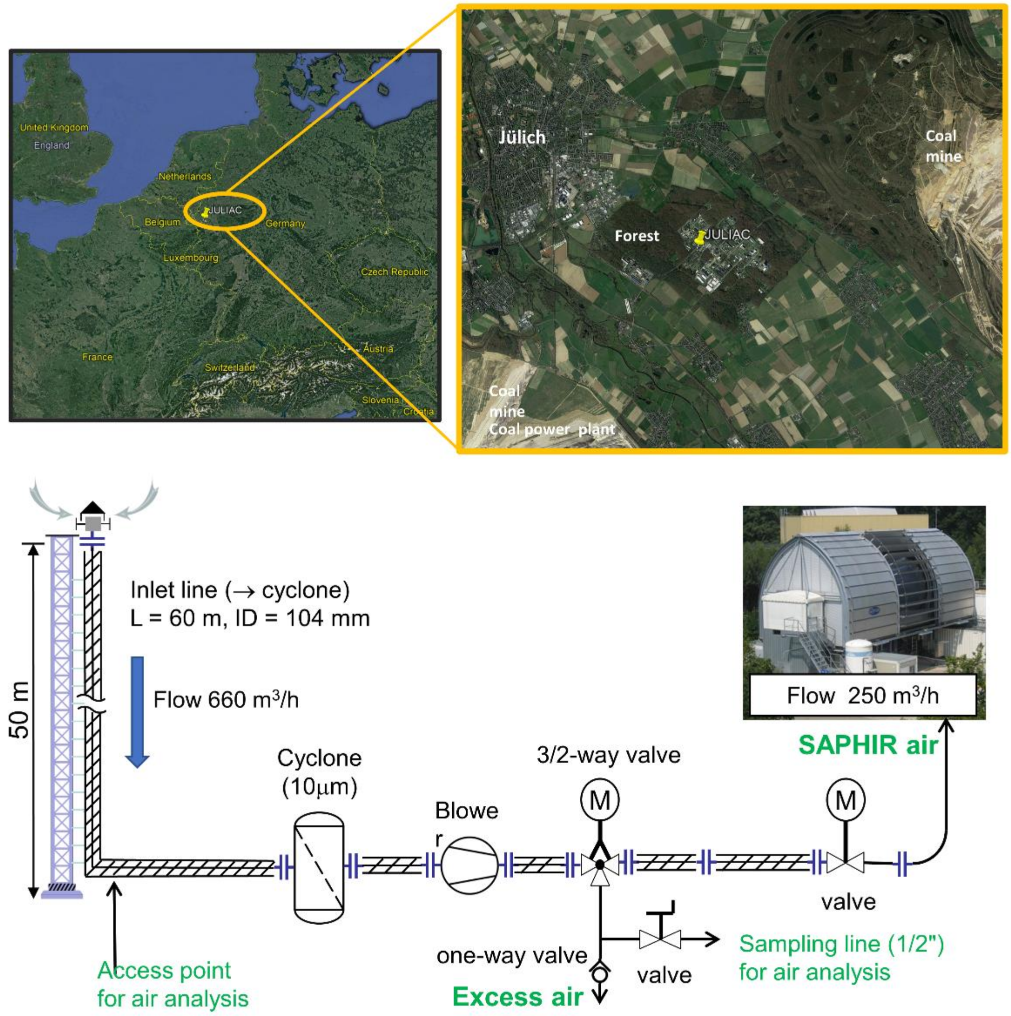

Supplementary Figure 24. The location and surrounding environment of the campaign site, as well as the basic experimental setup of the JULIAC campaign.

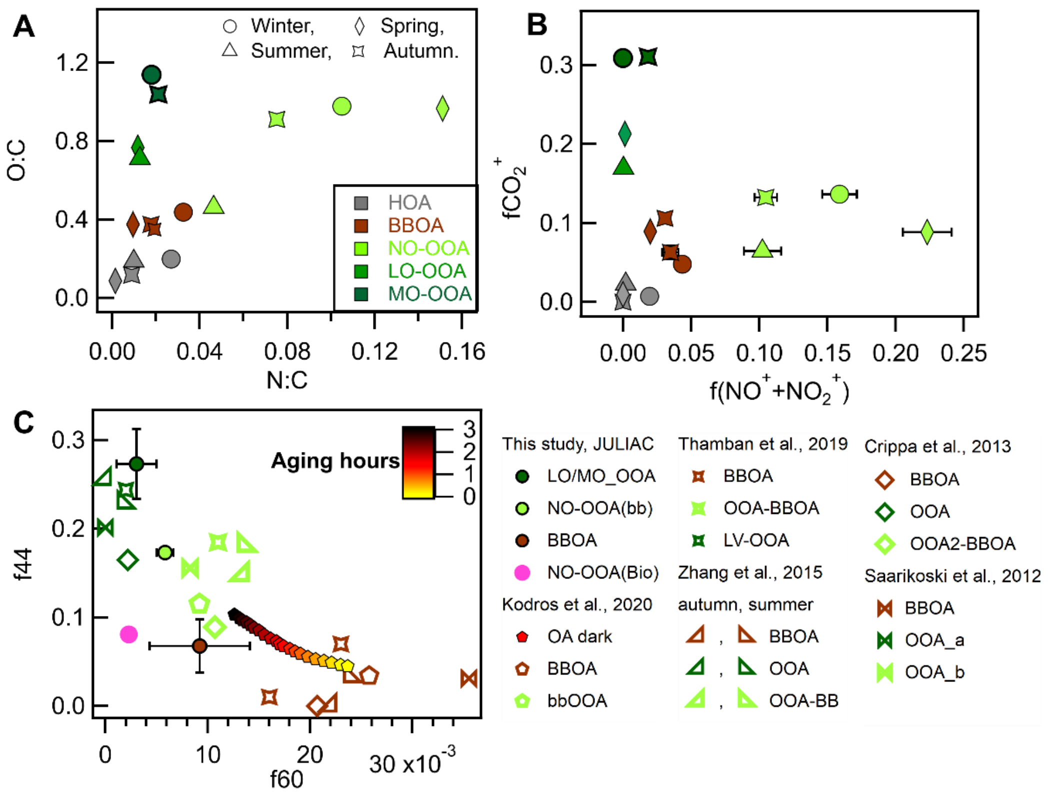

Supplementary Figure 25. Seasonal primary OA and secondary OOA resolved by the PMF analysis of measurements of aerosol nitrate and organics during the JULIAC campaign are shown as **(A)** elemental ratio O:C versus N:C, and **(B)** the framework of fragment fractions of CO_2^+ (fCO_2^+), and NO^++NO_2^+ ($f(NO^+ + NO_2^+)$). **(C)** OA factors resolved in this study are compared with that in other OA field and chamber studies as ratio of the integrated signal at m/z 44 and 60 to the total signal in the OA mass spectrum (f44 and f60) and the OA aging during the dark oxidation of biomass burning emission by $NO_3\cdot$ in chamber study [16].

Supplementary Table 1. Temperature-dependent reaction rate constants calculated by averaged night temperature (UTC 18:00-5:00) and NIST kinetics database (https://kinetics.nist.gov/kinetics/KineticsSearchForm.jsp).

Reaction	Seasons	Averaged temperature K (UTC 18:00-5:00)	Arrhenius equation	k(T) cm^3 $molecule^{-1}$ s^{-1}	reference
Furan+ NO_3·	Winter	278	k(T) = 1.30E-13 * exp (5.82/RT)	1.51E-12	[17]
	Spring	283		1.47E-12	
	Summer	293		1.39E-12	
	Autumn	277		1.51E-12	
Acetonitrile+ NO_3·	All seasons	---	---	5.00E-19	[18]
Phenol+ NO_3·	All seasons	---	---	3.64E-12	[18]
Isoprene+ NO_3·	Winter	278	k(T) = 3.02E-12 * exp (-3.71/RT)	5.65E-13	[18]
	Spring	283		5.92E-13	
	Summer	293		6.50E-13	
	Autumn	277		5.62E-13	
α-pinene+ NO_3·	Winter	278	k(T) = 1.19E-12 * exp (4.07/RT)	6.46E-12	[18]
	Spring	283		6.38E-12	
	Summer	293		6.21E-12	
	Autumn	277		6.47E-12	
β-pinene+ NO_3·	All seasons	298	---	2.51E-12	[18]
d-limonene+ NO_3·	All seasons	298	---	1.22E-11	[18]
Naphthalene+ NO_3·	All seasons	298	---	2.00E-11	[19]
Furan+ O_3	All seasons	---	---	2.42E-18	[20]
Isoprene+ O_3	Winter	278	k(T) = 5.60E-15 * exp (-15.05/RT)	7.73E-18	[21]
	Spring	283		8.83E-18	
	Summer	293		1.15E-17	
	Autumn	277		7.62E-18	
	Winter	278	k(T) = 4.80E-16* exp (-4.41/RT)	6.63E-17	[22]

α-pinene+ O_3	Spring	283		6.98E-17	
	Summer	293		7.75E-17	
	Autumn	277		6.59E-17	
β-pinene+ O_3	Winter	278	k(T) = 1.74E-15* exp (-10.78/RT)	1.53E-17	[22]
	Spring	283		1.69E-17	
	Summer	293		2.06E-17	
	Autumn	277		1.51E-17	
Limonene+ O_3	Winter	278	k(T) = 2.95E-15* exp (-6.51/RT)	1.64E-16	[22]
	Spring	283		1.76E-16	
	Summer	293		2.01E-16	
	Autumn	277		1.63E-16	
Naphthalene+ O_3	All seasons	295	---	2.81E-19	[23]

Supplementary Table 2. The relative importance of different oxidants ($NO_3\cdot$ and O_3) for biomass-burning VOCs (furan, naphthalene) and BVOCs(isoprene, α-pinene, β-pinene, and limonene) calculated by corresponding reaction rate constants and the average concentrations of $NO_3\cdot$ and O_3 between 18:00 and 5:00 UTC.

Species	Seasons	Averaged Night Conc (UTC 18:00-5:00)		Rate Constant cm^3 molecule^{-1} s^{-1}		Relative importance		Dark SOA yield from literature	
		$NO_3\cdot$ ppt	O_3 ppb	$NO_3\cdot$	O_3	$NO_3\cdot$	O_3	$NO_3\cdot$	O_3
isoprene	Winter	0.7	28.2	5.65E-13	7.73E-18	0.64	0.36	0.05-0.15 [24-26]	0.01-0.09 [27]
	Spring	1.7	38.5	5.92E-13	8.83E-18	0.75	0.25		
	Summer	4.7	36.6	6.5E-13	1.15E-17	0.88	0.12		
	Autumn	0.7	17.3	5.62E-13	7.62E-18	0.74	0.26		
α-pinene	Winter	0.7	28.2	6.46E-12	6.63E-17	0.71	0.29	0.007-0.25 [28-30]	0.15 [31]
	Spring	1.7	38.5	6.38E-12	6.98E-17	0.80	0.20		
	Summer	4.7	36.6	6.21E-12	7.75E-17	0.91	0.09		
	Autumn	0.7	17.3	6.47E-12	6.59E-17	0.79	0.21		
β-pinene	Winter	0.7	28.2	2.51E-12	1.53E-17	0.80	0.20	0.5-0.55 [14,32,33]	0.03-0.05 [34,35]
	Spring	1.7	38.5	2.51E-12	1.69E-17	0.87	0.13		
	Summer	4.7	36.6	2.51E-12	2.06E-17	0.94	0.06		
	Autumn	0.7	17.3	2.51E-12	1.51E-17	0.87	0.13		
limonene	Winter	0.7	28.2	1.22E-11	1.64E-16	0.65	0.35	0.44-2.31 [33,36]	0.24-0.55 [35,37]
	Spring	1.7	38.5	1.22E-11	1.76E-16	0.75	0.25		
	Summer	4.7	36.6	1.22E-11	2.01E-16	0.89	0.11		
	Autumn	0.7	17.3	1.22E-11	1.63E-16	0.74	0.26		
Furan	Winter	0.7	28.2	1.51E-12	2.42E-18	0.94	0.06	0.016-0.024 [38]	---
	Spring	1.7	38.5	1.47E-12	2.42E-18	0.96	0.04		
	Summer	4.7	36.6	1.39E-12	2.42E-18	0.99	0.01		
	Autumn	0.7	17.3	1.51E-12	2.42E-18	0.96	0.04		
Naphthalene	Winter	0.7	28.2	2E-11	2.81E-19	1.00	0.00	---	0.23-0.37 [39]
	Spring	1.7	38.5	2E-11	2.81E-19	1.00	0.00		
	Summer	4.7	36.6	2E-11	2.81E-19	1.00	0.00		
	Autumn	0.7	17.3	2E-11	2.81E-19	1.00	0.00		
Catechol	-	-	-	-	-	-	-	1.38-1.6 [40 41-43]	0.17-0.86 [40-43]

Supplementary Table 3. Correlation analysis between the concentration of BBOA and NO-OOA(bb) during winter, spring, and autumn for different levels of aerosol liquid water content. NO-OOA(bb)/BBOA is the slope of the linear fit, and R^2 is the correlation coefficient of the linear fit. The number fraction of dots represents the raw data number located in the corresponding ALWC range normalized to the total raw data number.

**ALWC** μg/m^3	**NO-OOA(bb)/BBOA**	**R^2**	**Dots number fraction**	**Error Y-axis** μg/m^3
0-10	0.83	0.63	90.3%	0.005
10-20	1.27	0.72	3.1%	0.038
20-30	1.62	0.71	1.1%	0.079
30-40	1.36	0.93	0.5%	0.045
40-50	1.31	0.95	0.5%	0.034
>50	1.72	0.90	4.5%	0.044

Supplementary Table 4. An overview of observed OOA with increases of uncertain origin during the night in previous field studies. Analysis methods and explanations of the enhancement of OOA during night are summarized as well.

Index	Ambiguous factors	Season and sites	Analysis methods	Explanations in cited studies	Citation
1	OOA2-BBOA	Winter, Paris	Levoglucosan time series correlation	A mixture of primary BBOA and background OOA	[44]
2	BBOA	Spring, Mexican Plateau	Profile correlation with photochemical aged BBOA in smog chamber aging experiment	A mixture of primary and secondary OA from biomass-burning emissions	[45]
3	Mixing factor biomass burning (BB)/SOA	Whole year, Hong Kong	Levoglucosan and 4-nitrocatechol, benzenetricarboxylic acids time series correlation	SOA from photo-oxidation of biomass burning emissions	[46]
4	Mixing factor LO-OOA/, MO-OOA	Whole year, Houston, Texas	Profile correlation of aqOOA factor, R=0.96	SOA mainly from aqueous-phase chemistry suggested	[47]
5	OOA	Winter, Athens	O:C 0.46, Clear midnight peak of OOA, Organic nitrate mass ratio diurnal pattern	SOA not from nitrate radical-derived oxidation since aerosol nitrates found during night were mainly inorganic	[12]
6	Aged-BBOA	Winter, Dongguan	Time-series correlation with $C_2H_4O_2^+$, R^2=0.71, O:C=0.60	SOA from biomass burning aging.	[48]
	OOA2	Winter, Bologna	Levoglucosan time series correlation R=0.7	Background-aged OA	[49]
7	Mixing factor LO-OOA	The whole year, Houston, Texas	Time-series correlation with ON, R=0.73, Profile correlation of aqOOA factor profile.	SOA from nocturnal NO_3-initiated oxidation of anthropogenic and biogenic VOCs, possibly aqueous phase chemistry.	[47]

8	Mixing factor LO-OOA	Summer, Southeastern United States	Time-series correlation with ON, R=0.81	SOA from NO_3-derived oxidation of Biogenic VOCs	[14]
9	91factor	Summer, Tennessee	----	SOA from both photochemistry and nighttime chemistry	[50]
10	OOA-3	Sping, Amazon Basin,	----	Fresh SOA from Biogenic VOCs oxidation combined with the effect of the development of the nocturnal boundary layer.	[13]

Supplementary Table 5. Comparison of results from the PMF analysis for just measurements of aerosol organics and the PMF analysis of measurements of both nitrate and organics for each season, to show the same source resolved in these two types of PMF analysis.

Org_PMF vs Org+NO_3 PMF				
Factor Correlation		t_series /R^2	Spectrum/ R^2	Spectrum (except NO^+&NO_2^+)/R^2
Winter	HOA	0.87	0.97	0.97
	BBOA	0.99	0.96	0.96
	NO-OOA(bb)	0.95	0.99	0.99
	MO-OOA	0.43	1	1
	Trans-OA	0.87	0.97	0.97
Spring	HOA	0.06	0.92	0.92
	BBOA	0.91	0.77	0.78
	NO-OOA(bb)	0.89	0.39	0.95
	LO-OOA	0.81	1	1
	Trans-OA	0.88	0	0.8
	MSA-OA	0.96	0.53	0.98
Summer	HOA	0.87	0.62	0.62
	MSA-OA	0.37	0.94	0.98
	NO-OOA(Bio)	0.95	0.67	0.99
	LO-OOA	0.99	1	1
Autumn	HOA	0.96	1	**1**
	BBOA	0.97	1	1
	BBOA2	0.89	0.99	0.99
	MO-OOA	0.96	1	1
	NO-OOA	0.97	0.99	0.99

Supplementary Table 6. Seasonal overview for the correlation of the time series of nocturnal OOA (NO-OOA) with gas, liquid, and particle phase tracers, including aerosol bulk nitrate (Bulk NO_3) and aerosol ion $C_2H_4O_2^+$ measured by HR-ToF-AMS, particulate organic nitrate and Hydroxymethanesulfonate (HMS) calculated based on HR-ToF-AMS measurements, primary biomass-burning OA (BBOA) resolved by PMF analysis, gas phase CO, furan, and aerosol liquid water content (ALWC)

Correlation, R^2	NO-OOA(bb), winter	NO-OOA(bb), spring	NO-OOA(Bio), summer	NO-OOA(bb), fall
Organic nitrate	0.28	0.59	0.83	0.60
Bulk nitrate	0.22	0.04	0.48	0.37
$C_2H_4O_2^+$	0.68	0.82	0.70	0.73
Gas CO	0.44	0.48	0.64	0.75
BBOA	0.50	0.62	---	0.48
Furan	0.32	0.49	0.01	---
HMS	0.13	0.21	0.23	0.44
ALWC	0.01	0.01	0.04	0.01

Supplementary References

1 Bohn, B. & Zilken, H. Model-aided radiometric determination of photolysis frequencies in a sunlit atmosphere simulation chamber. *Atmos. Chem. Phys.* **5**, 191-206 (2005).

2 Bohn, B., Rohrer, F., Brauers, T. & Wahner, A. Actinometric measurements of NO_2 photolysis frequencies in the atmosphere simulation chamber SAPHIR. *Atmos. Chem. Phys.* **5**, 493-503 (2005).

3 Tan, Z. *et al.* Seasonal variation of nitryl chloride and its relation to gas-phase precursors during the JULIAC campaign in Germany. *Atmos. Chem. Phys. Discuss.* **2022**, 1-30 (2022).

4 Cho, C. *et al.* Characterization of a chemical modulation reactor (CMR) for the measurement of atmospheric concentrations of hydroxyl radicals with a laser-induced fluorescence instrument. *Atmospheric Measurement Techniques* **14**, 1851-1877 (2021).

5 Drewnick, F. *et al.* A New Time-of-Flight Aerosol Mass Spectrometer (TOF-AMS)—Instrument Description and First Field Deployment. *Aerosol Sci Tech* **39**, 637-658 (2005).

6 Jimenez, J. L. *et al.* Ambient aerosol sampling using the Aerodyne Aerosol Mass Spectrometer. *Journal of Geophysical Research: Atmospheres* **108** (2003).

7 Middlebrook, A. M., Bahreini, R., Jimenez, J. L. & Canagaratna, M. R. Evaluation of Composition-Dependent Collection Efficiencies for the Aerodyne Aerosol Mass Spectrometer using Field Data. *Aerosol Sci Tech* **46**, 258-271 (2012).

8 Wagner, N. L. *et al.* Diode laser-based cavity ring-down instrument for NO_3, N_2O_5, NO, NO_2 and O_3 from aircraft. *Atmos. Meas. Tech.* **4**, 1227-1240 (2011).

9 Visser, S. *et al.* Advanced source apportionment of size-resolved trace elements at multiple sites in London during winter. *Atmos. Chem. Phys.* **15**, 11291-11309 (2015).

10 Kostenidou, E., Lee, B.-H., Engelhart, G. J., Pierce, J. R. & Pandis, S. N. Mass Spectra Deconvolution of Low, Medium, and High Volatility Biogenic Secondary Organic Aerosol. *Environ Sci Technol* **43**, 4884-4889 (2009).

11 Saarikoski, S. *et al.* Chemical characterization of springtime submicrometer aerosol in Po Valley, Italy. *Atmos Chem Phys* **12**, 8401-8421 (2012).

12 Florou, K. *et al.* The contribution of wood burning and other pollution sources to wintertime organic aerosol levels in two Greek cities. *Atmos Chem Phys* **17**, 3145-3163 (2017).

13 Chen, Q. *et al.* Submicron particle mass concentrations and sources in the Amazonian wet season (AMAZE-08). *Atmos Chem Phys* **15**, 3687-3701 (2015).

14 Xu, L. *et al.* Effects of anthropogenic emissions on aerosol formation from isoprene and monoterpenes in the southeastern United States. *Proc Natl Acad Sci U S A* **112**, E4506-4507 (2015).

15 Paglione, M. *et al.* Identification of humic-like substances (HULIS) in oxygenated organic aerosols using NMR and AMS factor analyses and liquid chromatographic techniques. *Atmos. Chem. Phys.* **14**, 25-45 (2014).

16 Kodros, J. K. *et al.* Rapid dark aging of biomass burning as an overlooked source of oxidized organic aerosol. *Proc Natl Acad Sci U S A* **117**, 33028-33033 (2020).

17 Cabañas, B. *et al.* Oxidation of Heterocycles in the Atmosphere: Kinetic Study of Their Reactions with NO3 Radical. *The Journal of Physical Chemistry A* **108**, 10818-10823 (2004).

18 Atkinson, R. Kinetics and Mechanisms of the Gas‐Phase Reactions of the NO_3 Radical with Organic Compounds. *Journal of Physical and Chemical Reference Data* **20**, 459-507 (1991).

19 Atkinson, R. & Arey, J. Mechanisms of the gas-phase reactions of aromatic hydrocarbons and PAHS with OH and NO_3 radicals. *Polycyclic Aromatic Compounds* **27**, 15-40 (2007).

20 Atkinson, R., Aschmann, S. M. & Carter, W. P. L. Kinetics of the reactions of O_3 and OH radicals with furan and thiophene at 298 ± 2 K. *International Journal of Chemical Kinetics* **15**, 51-61 (1983).

21 Grosjean, E. & Grosjean, D. Rate constants for the gas-phase reaction of ozone with 1, 1-disubstituted alkenes. *International Journal of Chemical Kinetics* **28**, 911-918 (1996).

22 Khamaganov, V. G. & Hites, R. A. Rate Constants for the Gas-Phase Reactions of Ozone with Isoprene, α- and β-Pinene, and Limonene as a Function of Temperature. *The Journal of Physical Chemistry A* **105**, 815-822 (2001).

23 Atkinson, R. & Aschmann, S. M. Kinetics of the reactions of naphthalene, 2-methylnaphthalene, and 2,3-dimethylnaphthalene with OH radicals and with O_3 at 295 ± 1 K. *International Journal of Chemical Kinetics* **18**, 569-573 (1986).

24 Brownwood, B. *et al.* Gas-Particle Partitioning and SOA Yields of Organonitrate Products from NO_3-Initiated Oxidation of Isoprene under Varied Chemical Regimes. *ACS Earth Space Chem* **5**, 785-800 (2021).

25 Ng, N. L. *et al.* Secondary organic aerosol (SOA) formation from reaction of isoprene with nitrate radicals (NO_3). *Atmos. Chem. Phys.* **8**, 4117-4140 (2008).

26 Rollins, A. W. *et al.* Isoprene oxidation by nitrate radical: alkyl nitrate and secondary organic aerosol yields. *Atmos. Chem. Phys.* **9**, 6685-6703 (2009).

27 Clark, C. H. *et al.* Temperature Effects on Secondary Organic Aerosol (SOA) from the Dark Ozonolysis and Photo-Oxidation of Isoprene. *Environ Sci Technol* **50**, 5564-5571 (2016).

28 Hallquist, M., Wängberg, I., Ljungström, E., Barnes, I. & Becker, K. Aerosol and Product Yields from NO_3 Radical-Initiated Oxidation of Selected Monoterpenes. *Environ Sci Technol* **33**, 553-559 (1999).

29 Mutzel, A. *et al.* Importance of secondary organic aerosol formation of α-pinene, limonene, and m-cresol comparing day- and nighttime radical chemistry. *Atmos Chem Phys* **21**, 8479-8498 (2021).

30 Day, D. A. *et al.* Secondary Organic Aerosol Mass Yields from NO3 Oxidation of α-Pinene and Δ-Carene: Effect of RO2 Radical Fate. *The Journal of Physical Chemistry A* **126**, 7309-7330 (2022).

31 Shilling, J. E. *et al.* Particle mass yield in secondary organic aerosol formed by the dark ozonolysis of α-pinene. *Atmos. Chem. Phys.* **8**, 2073-2088 (2008).

32 Fry, J. L. *et al.* Organic nitrate and secondary organic aerosol yield from NO_3 oxidation of β-pinene evaluated using a gas-phase kinetics/aerosol partitioning model. *Atmos. Chem. Phys.* **9**, 1431-1449 (2009).

33 Boyd, C. M., Nah, T., Xu, L., Berkemeier, T. & Ng, N. L. Secondary Organic Aerosol (SOA) from Nitrate Radical Oxidation of Monoterpenes: Effects of Temperature, Dilution, and Humidity on Aerosol Formation, Mixing, and Evaporation. *Environ Sci Technol* **51**, 7831-7841 (2017).

34 Griffin, R. J., Cocker III, D. R., Flagan, R. C. & Seinfeld, J. H. Organic aerosol formation from the oxidation of biogenic hydrocarbons. *Journal of Geophysical Research: Atmospheres* **104**, 3555-3567 (1999).

35 Zhao, D. F. *et al.* Secondary organic aerosol formation from hydroxyl radical oxidation and ozonolysis of monoterpenes. *Atmos Chem Phys* **15**, 991-1012 (2015).

36 Fry, J. L. *et al.* Secondary organic aerosol formation and organic nitrate yield from NO_3 oxidation of biogenic hydrocarbons. *Environ Sci Technol* **48**, 11944-11953 (2014).

37 Zhang, J., Huff Hartz, K. E., Pandis, S. N. & Donahue, N. M. Secondary Organic Aerosol Formation from Limonene Ozonolysis: Homogeneous and Heterogeneous Influences as a Function of NOx. *The Journal of Physical Chemistry A* **110**, 11053-11063 (2006).

38 Joo, T., Rivera-Rios, J. C., Takeuchi, M., Alvarado, M. J. & Ng, N. L. Secondary Organic Aerosol Formation from Reaction of 3-Methylfuran with Nitrate Radicals. *ACS Earth and Space Chemistry* **3**, 922-934 (2019).

39 Riva, M. *et al.* Gas and particulate phase products from the ozonolysis of acenaphthylene. *Atmospheric Environment* **142**, 104-113 (2016).

40 Finewax, Z., de Gouw, J. A. & Ziemann, P. J. Identification and Quantification of 4-Nitrocatechol Formed from OH and NO_3 Radical-Initiated Reactions of Catechol in Air in the Presence of NO_x: Implications for Secondary Organic Aerosol Formation from Biomass Burning. *Environ Sci Technol* **52**, 1981-1989 (2018).

41 Fredrickson, C. D. *et al.* Formation and Evolution of Catechol-Derived SOA Mass, Composition, Volatility, and Light Absorption. *ACS Earth and Space Chemistry* **6**, 1067-1079 (2022).

42 Coeur-Tourneur, C. *et al.* Aerosol formation yields from the reaction of catechol with ozone. *Atmospheric Environment* **43**, 2360-2365 (2009).

43 Coeur-Tourneur, C., Foulon, V. & Laréal, M. Determination of aerosol yields from 3-methylcatechol and 4-methylcatechol ozonolysis in a simulation chamber. *Atmospheric Environment* **44**, 852-857 (2010).

44 Crippa, M. *et al.* Wintertime aerosol chemical composition and source apportionment of the organic fraction in the metropolitan area of Paris. *Atmos Chem Phys* **13**, 961-981 (2013).
45 DeCarlo, P. F. *et al.* Investigation of the sources and processing of organic aerosol over the Central Mexican Plateau from aircraft measurements during MILAGRO. *Atmos Chem Phys* **10**, 5257-5280 (2010).
46 Cheng, Y., Ma, Y. & Hu, D. Tracer-based source apportioning of atmospheric organic carbon and the influence of anthropogenic emissions on secondary organic aerosol formation in Hong Kong. *Atmos. Chem. Phys.* **21**, 10589-10608 (2021).
47 Dai, Q. *et al.* Seasonal differences in formation processes of oxidized organic aerosol near Houston, TX. *Atmos Chem Phys* **19**, 9641-9661 (2019).
48 Zhu, Q. *et al.* Improved source apportionment of organic aerosols in complex urban air pollution using the multilinear engine (ME-2). *Atmospheric Measurement Techniques* **11**, 1049-1060 (2018).
49 Gilardoni, S. *et al.* Direct observation of aqueous secondary organic aerosol from biomass-burning emissions. *Proc Natl Acad Sci U S A* **113**, 10013-10018 (2016).
50 Budisulistiorini, S. H. *et al.* Examining the effects of anthropogenic emissions on isoprene-derived secondary organic aerosol formation during the 2013 Southern Oxidant and Aerosol Study (SOAS) at the Look Rock, Tennessee ground site. *Atmos Chem Phys* **15**, 8871-8888 (2015).
